# Transmission Dynamics of Visceral Leishmaniasis in the Indian Subcontinent – A Systematic Literature Review

**DOI:** 10.1371/journal.pntd.0004896

**Published:** 2016-08-04

**Authors:** Siddhivinayak Hirve, Marleen Boelaert, Greg Matlashewski, Dinesh Mondal, Byron Arana, Axel Kroeger, Piero Olliaro

**Affiliations:** 1 Global Influenza Programme, World Health Organization, Geneva, Switzerland; 2 Epidemiology and Control of Tropical Diseases, Institute of Tropical Medicine, Antwerp, Belgium; 3 Department of Microbiology and Immunology, McGill University, Montreal, Canada; 4 Nutrition and Clinical Services Division, International Center for Diarrheal Disease Research, Dhaka, Bangladesh; 5 Drugs for Neglected Diseases Initiative, Geneva, Switzerland; 6 Special Programme on Research and Training in Tropical Diseases, World Health Organization, Geneva, Switzerland; Institute of Postgraduate Medical Education and Research, INDIA

## Abstract

**Background:**

As Bangladesh, India and Nepal progress towards visceral leishmaniasis (VL) elimination, it is important to understand the role of asymptomatic Leishmania infection (ALI), VL treatment relapse and post kala-azar dermal leishmaniasis (PKDL) in transmission.

**Methodology/ Principal Finding:**

We reviewed evidence systematically on ALI, relapse and PKDL. We searched multiple databases to include studies on burden, risk factors, biomarkers, natural history, and infectiveness of ALI, PKDL and relapse. After screening 292 papers, 98 were included covering the years 1942 through 2016. ALI, PKDL and relapse studies lacked a reference standard and appropriate biomarker. The prevalence of ALI was 4–17-fold that of VL. The risk of ALI was higher in VL case contacts. Most infections remained asymptomatic or resolved spontaneously. The proportion of ALI that progressed to VL disease within a year was 1.5–23%, and was higher amongst those with high antibody titres. The natural history of PKDL showed variability; 3.8–28.6% had no past history of VL treatment. The infectiveness of PKDL was 32–53%. The risk of VL relapse was higher with HIV co-infection. Modelling studies predicted a range of scenarios. One model predicted VL elimination was unlikely in the long term with early diagnosis. Another model estimated that ALI contributed to 82% of the overall transmission, VL to 10% and PKDL to 8%. Another model predicted that VL cases were the main driver for transmission. Different models predicted VL elimination if the sandfly density was reduced by 67% by killing the sandfly or by 79% by reducing their breeding sites, or with 4–6y of optimal IRS or 10y of sub-optimal IRS and only in low endemic setting.

**Conclusion/ Significance:**

There is a need for xenodiagnostic and longitudinal studies to understand the potential of ALI and PKDL as reservoirs of infection.

## Author Summary

The role of asymptomatic Leishmania infection (ALI), PKDL and VL relapse in transmission is unclear as VL elimination is achieved in the Indian subcontinent. ALI, PKDL and relapse studies lacked a reference standard and appropriate biomarker. ALI was 4–17-fold more prevalent than VL. The risk of ALI was higher in VL case contacts. Most infections remained asymptomatic or resolved spontaneously. The natural history of PKDL showed variability. Twenty nine percent had no past history of VL treatment. The risk of VL relapse was higher with HIV co-infection. Modelling studies predicted different effects. Early diagnosis was unlikely to eliminate VL in the long term. ALI was predicted to contribute to 82% of the overall transmission, VL to 10% and PKDL to 8%. Another model predicted that VL cases were the main driver for transmission. VL elimination was predicted if the sandfly density was reduced by 67% by killing the sandfly or by 79% by reducing their breeding sites, or with 4–6y of optimal IRS or 10y of sub-optimal IRS and only in low endemic setting. There is a need for more studies to fully understand the potential of ALI and PKDL as reservoirs of infection.

## Introduction

The concomitance of anthroponotic transmission of visceral leishmaniasis (VL), a single species of sandfly as the only known vector for transmission, the largely localized geographic endemicity of the disease, the availability of field-based diagnostic tests and highly effective drugs for treating VL, together, favour the elimination of the disease as a public health problem in the Indian subcontinent through effective surveillance, early detection and treatment, and integrated vector control strategies [1]. Furthermore, historical evidence of near-eradication of VL in the 1970s following insecticide spraying for malaria control in the 1950s and 1960s in India supports the rationale for VL elimination in the Indian subcontinent [2]. In 2005, the Governments of Bangladesh, India and Nepal signed a memorandum of understanding to eliminate VL and set the target to reduce its annual incidence to less than 1 per 10,000 population (at the upazila level in Bangladesh, block level in India and district level in Nepal) by 2015 [3]. This political commitment was recently reinforced at a meeting of the Ministers of Health in September 2014 with the aim to make the Southeast Asia region including Bhutan and Thailand free of VL by 2017 or earlier [4]. Substantial progress has been made towards the elimination target in Bangladesh with only two out of 98 endemic upazilas reporting a incidence rate greater than 1 per 10,000 in 2015 (Table A in S1 Text). An external assessment of the national VL control program in Nepal conducted in 2015 indicated that all the 12 previously endemic districts have achieved the elimination target (Table B in S1 Text). On the other hand, despite a declining trend in the number of reported VL cases, 90 out of 456 blocks continue to report an annual incidence of more than 1 per 10,000 in India (Table C in S1 Text). Despite substantial progress, a major challenge evident from recent outbreak investigations and surveillance data has been the increasing emergence of new ecological niches of indigenous transmission in previously non-endemic regions of Bangladesh and Nepal [5–9].

Research on VL and post Kala-azar dermal leishmaniasis (PKDL) has focused largely on the clinical and epidemiology aspects of the disease. A large body of research has evaluated diagnostics [10–13], potential biomarkers for treatment response of VL and PKDL [14], treatment options [15], and vector control [16,17]. The parasite, vector species and alternative animal reservoirs for VL infection in Africa differ from that in the Indian subcontinent and research findings cannot be simply applied from one to the other [18]. Many questions remain about the natural history, the progression of asymptomatic Leishmania infection (ALI) to symptomatic VL disease, development of PKDL, the pathogenesis, the immune response to infection and disease [19]. Moreover, data on transmission dynamics, infectiveness and vector bionomics, role and duration of acquired immunity after infection are scarce, which limits the use of mathematical modelling to predict and inform treatment and vector control strategies for VL elimination [20]. Furthermore, the complex interactions of co-infection with HIV alters the transmission dynamics and increases the vulnerability of both infections to treatment failure and relapse and has the potential to thwart elimination efforts [21–25]. The emergence of parasite resistance to antimonials that led to a sharp increase of up to 65% treatment failure in a case series [26–28] seen in Bihar, India between 1980 and 1997, also suggested the potential for development of resistance to miltefosine [29–31] and liposomal amphotericin B [32,33]. This could further alter the transmission dynamics and is a major concern for elimination efforts [34,35].

As countries progress towards the elimination target using current strategies of early detection and treatment of clinical disease and vector control, it is necessary to understand the consequences of under-reporting on planning and evaluating VL elimination strategies, the contribution of ALI to sustain transmission and emergence of new hotspots for infection [36]. It is equally important to understand the contribution of PKDL to transmission and its potential role as a reservoir of infection, to inform how long elimination efforts need to be continued and how they should be targeted to prevent recrudescence of new VL epidemics in the future [1]. The objective of this systematic review was to synthesize existing literature on transmission dynamics and relapse rates of VL caused by *L donovani* in the Indian subcontinent. In particular, the review focused on the role of ALI and PKDL as potential reservoirs for transmission so as to inform current strategies for achieving and maintaining VL elimination in the Indian subcontinent.

## Methods

**Fig 1:**
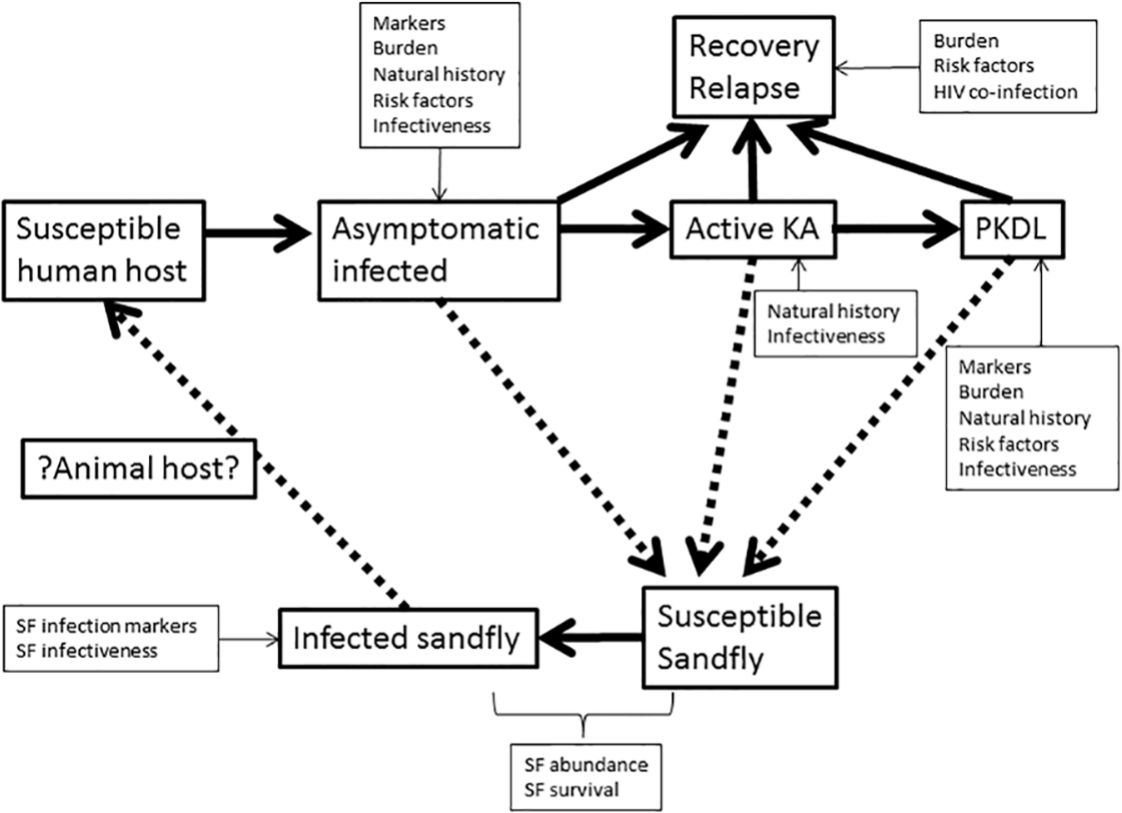
Conceptual framework for the systematic review of transmission dynamics of visceral leishmaniasis in the Indian subcontinent. For simplicity, the relapse and recovery stages are shown together and the infectiveness of the relapse stage to the sandfly is omitted.

The Preferred Reporting Items for Systematic Reviews and Meta-analysis (PRISMA) statement was used to guide the process and reporting of this review (Table D in S1 Text) [37]. The broader outcome of interest was to systematically review information related to and affecting transmission dynamics of visceral leishmaniasis from the perspective of elimination. Primary outcomes reviewed were the role of ALI and PKDL as potential reservoirs and their contribution to transmission. Secondary outcomes of interest included the potential of HIV co-infection and relapse in altering the transmission dynamics, and vector bionomics related to transmission (Fig 1).

We searched multiple databases (United States National Library of Medicine, Cochrane Library, WHO Library Information System, National Database of Indian Medical Journals) using different combinations of MeSH terms (with synonyms and closely related words) such as ‘Leishmaniasis, Visceral’, ‘Disease transmission, Infectious’, ‘risk factors’, ‘relapse’, ‘recurrence’, ‘asymptomatic infections’, ‘Basic reproduction number’, ‘disease outbreaks’, ‘contact tracing’, and text words such as ‘transmission risk’, ‘disease progression’, ‘surveillance’ etc. with and without restriction to MeSH terms ‘Bangladesh OR India OR Nepal’. An example of a search query is shown in Table E in S1 Text. Duplicates were removed and two reviewers (SH, SQ) independently screened the title and abstract of all articles and determined eligibility. We also screened articles that showed up as related during the search. The full text of all eligible articles was further assessed by SH and SQ independently for inclusion. We also screened the references of all included articles to identify any new articles hitherto missed. We reviewed all the earlier reviews and the individual studies that were included in the past reviews. Articles were included based on consensus between SH and SQ. For multiple articles referring to the same study, we included the article with the most recent finding. However, we included all articles that referred to different aspects of the same study. The research community involved in visceral leishmaniasis research in the Indian subcontinent is small and well-networked. We requested researchers for information on ongoing studies and share preliminary findings or manuscripts in preparation or submission. We accessed meeting reports of the Regional Technical Advisory Group (RTAG) for Kala-azar elimination, research study findings that were presented at recent conferences and presentations made by country program managers at a WHO TDR meeting for the most recent update on VL status on the Indian subcontinent. We could not assess the extent of publication bias if any.

The scope and eligibility criteria for the review are given in Box 1.

**Box 1**

We included studies on VL and PKDL:

- from Bangladesh, Bhutan, India and Nepal
- articles in the English language
- articles related to the burden of ALI and PKDL–their markers (excluding genetic markers) and risk factors
- related to the natural history and transmission dynamics of VL–the infectiveness of the ALI and PKDL
- on co-infection with HIV and relapse following VL and PKDL

We excluded studies on VL and PKDL that focused solely on:

- burden, clinical aspects and epidemiology of VL and outbreak investigations
- evaluated diagnostics
- clinical drug trials (except to estimate relapse rate)
- evaluated early detection, treatment and vector control program strategies

The following topics were beyond the scope of this systematic review:

- pathogenesis and immunogenicity of VL and PKDL
- bionomics of the *Ph*. *argentipes* vector
- mother to child transmission and transmission through blood transfusion

### Mathematical modelling of VL transmission

Data was extracted from the full text articles directly into a structured table under variables such as diagnostic used, seroprevalence, negative sero-conversion, asymptomatic to symptomatic ratio, risk factors / markers for ALI, progression to symptomatic VL disease, relapse, risk factors for relapse, infectiveness, etc.

Simple proportions and risks (hazard ratio, risk ratio, odds ratio) as applicable and their range across different studies were used to describe the outcome variables of interest. We also compared the different mathematical models used for quantifying transmission dynamics with respect to their structures, data sources, assumptions, limitations, and predictions. We did not attempt a meta-analysis as the number of studies that focused on transmission dynamics, infectiveness were limited in the context of the Indian subcontinent. The risk of bias was ascertained using the Newcastle-Ottawa bias assessment scale for observational studies [38] and the Cochrane risk of bias assessment tool for trials [39].

## Results

**Fig 2:**
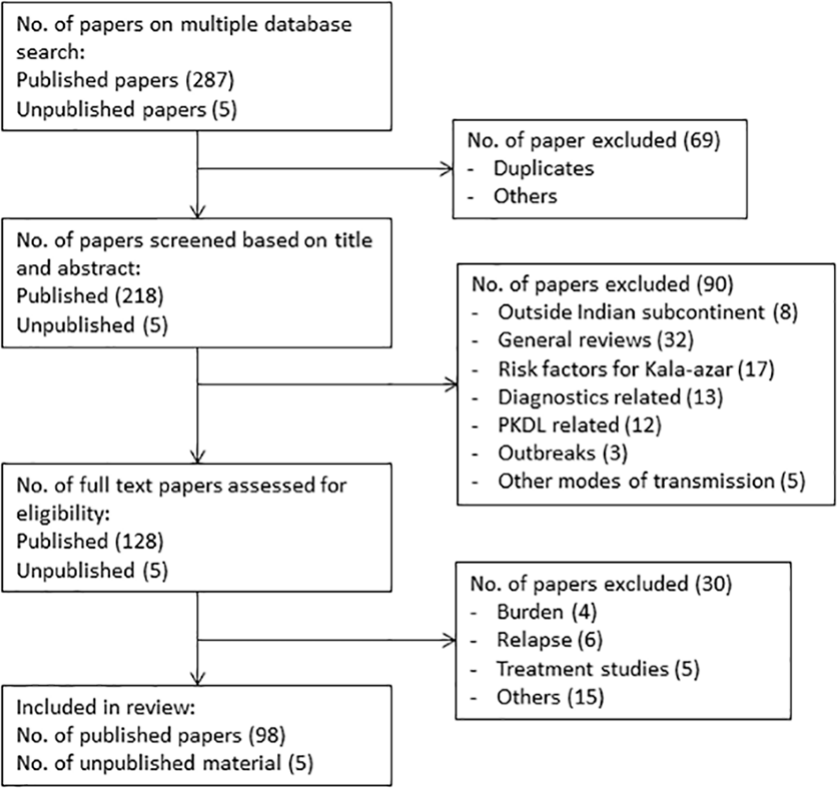
PRISMA flowchart of inclusion and exclusion of articles for the review.

The review covers Bangladesh, Bhutan, India and Nepal representing more than 73% of the global burden of VL in 2012 [40]. A total of 292 papers (including 5 meeting reports) were identified based on the multiple database searches as of 01 March 2016 of which 69 were excluded as duplicates or for other reasons. A further 90 papers were excluded (32 general reviews, 8 papers from outside the Indian subcontinent, 17 papers on risk factors for VL etc.) based on screening of the title and abstract. Another 30 papers were excluded (four were related to disease burden, six related to relapse etc.) after a full text appraisal. A total of 98 published and 5 meeting reports from 1942 to 2016 were included in the final review (Fig 2). The assessment of the risk of bias in the included studies is shown in Tables F to I in S1 Text. Most cross-sectional surveys did not justify sample size or demonstrated comparability of non-respondents or comparability of the different outcome groups. The risk of bias due to loss to follow up was high from most cohort studies. Case-control studies did not report on the non-response rates.

### Asymptomatic Leishmania infection–burden, risk factors, natural history, infectiveness

A total of 31 articles including two reviews were identified. ALI lacked a reference standard and appropriate biomarker. It was variably ascertained by a positive serology test (rk39 ICT, rk39 ELISA or DAT), PCR, qPCR or LST in an otherwise healthy individual from an endemic area [41,42]. Only one study established the specificity of the assay on non-endemic controls [43]. Individuals with past history of VL were often but not always excluded from serological surveys. Table 1 gives the prevalence and incidence of ALI by country and by the different tests and thresholds used to ascertain infection. The majority of studies from which prevalence was estimated were not population based. Seropositivity among endemic healthy controls in diagnostic evaluation studies were also included for estimating prevalence of ALI. The seropositivity as measured by antibody response to rk39 antigen was 7.4% [44]. The seropositivity as measured by antibody response to the saliva antigen of the sandfly vector ranged between 43.5–63.2%; this is a proxy for human exposure to sandfly but not necessarily infection [45]. The prevalence of ALI was 34.8% and 3.8% for a parasitaemia of >0 and >1 parasite genome/mL on qPCR respectively [46]. The prevalent ALI cases outnumbered that of prevalent symptomatic VL cases by a factor of 4.0 in Bangladesh, 13 in Nepal and ranging from 7.6 to 17 in India [47,48]. The ratio of incident asymptomatic infection to incident clinical disease increased with decreasing incidence rates of VL (Table J in S1 Text). However, more standardized and validated tests are needed to establish more accurately the prevalence of ALIs [46,49]. The risk of ALI was significantly higher (OR ranging from 1.25–5.5) in individuals in close contact (household member) with a known VL case [50,51] or with the presence of other seropositive or recently sero-converted individuals in the household (OR 1.37–2.22) [52] indicative of spatial and temporal clustering of infections [49,53]. Livestock ownership was associated with a lower risk for infection (OR 0.4–1.0) [54] in Nepal but a significantly higher risk (OR 1.16–2.1) in India [55,56]. In contrast, a higher cattle density in the community had a protective effect against infection in Bangladesh (OR 0.97) and Nepal (OR 0.63) [47,51]. Risk factors associated with VL such as poverty, low socioeconomic status, malnutrition, poor housing conditions, damp floors, mud walls, sleeping on the floor, sleeping outside and proximity to water bodies, also significantly increased the risk for ALI [51,52,55,57].

**Table 1:** ALI as a potential reservoir for transmission in the Indian subcontinent.

	Reference	Bangladesh	India	Nepal
**Prevalence of ALI**				
*- rk39 ICT*	[50,58–62]	0.25%	5.6–13.8%	
*- rk39 ELISA*	[41,42,44,47,49,63,64]	10–14%	5.4–26.3%	6.4–12.6%
*- DAT*	[41,42,44,50,51,54,56,61–63,65–72]	3.02%	3.1–26.4%	4.4–16.2%
*- LST*	[47,54,73,74]	19–35%	19–23.1%	13.2%
*- PCR*	[42,53,58,62,65,71,75,76]		7.2–36.9%	5.1–17.9%
**Prevalence among healthy contacts of current or past VL cases**				
*- rk39 ICT*	[60]	0.08%		
*- rk39 ELISA*	[42,77]		19.5–43.6%	
*- DAT*	[42,65,66,69]		14.4–100%	20.8%
*- IFAT*	[78]		17.5%	
*- LST*	[78]		5%	
*- PCR*	[42,65]		20.6%	12.5%
**Incidence (1y)**				
*- rk39 ICT*	[60]	0% (6mo)		
*- rk39 ELISA*	[41,44,46,47]	6.31%	1.3–27.3%	
*- DAT*	[41,44,46,48,55,72]		2.5–27.3%	2.9–7.2%
*- qPCR (parasite/ml)*	[46]		34.8% (>0); 3.8% (>1p/ml); 1.36% (>5p/ml);	
*-rKRP42 ELISA (at 30mo)*	[79]	23.4%		
**ALI to VL ratio**	[47,48]	4:1	7.6:1	9.6:1
**Risk factors for ALI**				
*- contact of VL (cf non-contact of VL) OR*	[47,50–52,57,65]	1.37–1.85	1.25–3.71	1.66–5.5
*- presence of other seropositive or sero-converter in house OR*	[52]		1.37–2.22	1.37–2.22
*- family size OR*	[54]			4.4
*- damp floor OR*	[55]		2.4	
*- mud walls OR*	[54,55]	28.9	4.3	3.0
*- proximity to pond OR*	[54,55]		2.1	3.7
*- proximity to forest OR*	[57]			3.67
*- livestock ownership OR*	[54–56]		1.16–2.1	0.4–1.0
*- cattle density OR*	[47,51]	0.97		0.63
*- spatial clustering of PCR+ human with PCR+ animals*	[53]			significant
*- Risk of spatial clustering of PCR+ or sero+ human for transmission*	[49,53]	High risk		No effect
*- sleeping covered/inside OR*	[55]		0.5–0.6	
*- age OR*	[41,47,51,52,54,56]	1.12	1.57–3.68	1.57–7.29
*- highest (cf lowest) SES OR*	[52]		0.63	0.63
*- poverty OR*	[51]			3.86
*- indoor residual spraying OR*	[55]		No effect	
*- IRS more than 18mo ago OR*	[52]		1.53	1.53
*- use of bednet OR*	[47,52,55]	No effect– 0.82 (ns)	0.7–1.09 (ns)	0.66 (ns)
**Proportion asymptomatic with spontaneous resolution at 1y**				
*- rk39 ELISA*	[41,47,62]	50.21%	59–60%	
*- DAT*	[41,48,62,66]		33–86.7%	86.7%
**Progression of ALI to VL within 1y**				
*- rk39 ICT*	[58,62]		12.5–23.1%	
*- rk39 ELISA*	[44,47,49]	5.4–25% (6mo– 2y)	1.8–23.3%	7.7%
*- DAT*	[44,48,50,68]	5.4% (2y)	1.5–16.6%	1.68^1^ – 9.8%
*- PCR*	[46,58]		2.5–17.9%	
*-rKRP42 ELISA*	[79]	29.2%		
*- Contacts of VL case*	[58,66,77]		24.1–69.1%	
*- Contacts of active VL case*	[66]		18.8%	
*- Contacts of cured VL case*	[66]		30.8%	
*- Non-contacts*	[58,66]		5–38%	
**Risk factors for progression of ALI to VL**				
*- contact of VL (cf non-contact) OR*	[47,62,66]	2.85	3.36–4.82	
*- contact of cured VL (cf contact of active VL) OR*	[66]		1.64	
*- Risk of spatial clustering of PCR+ or sero+ for progression to VL*	[49]	Highest risk		
*- age (10y increment) OR*	[47]	0.74		
*- beef*, *goat consumption OR*	[47]	0.49		
**Biomarkers for progression of ALI to VL**				
*- rk39 titres HR*	[44,80]			
*Negative*		reference	reference	reference
*Mod positive*		1.6	1.6–4.9	--
*Strongly positive*		17.7	7.7–39.6	26.9
*- rk39 titres among contacts of VL (positive predictive value)*	[77]		44% at 3mo, 56.6% at 6mo;	
*- rk39 / DAT titres high*	[46]		60%	
*- Sero-converters rk39 titres HR*	[44,80]			
*Negative*		reference	reference	
*Mod positive*		4.7	0.9 (ns)	
*Strongly positive*		165	15.9–123.9	
*- DAT titres HR*	[44]			
*<1*:*1600*			reference	reference
*1600 - <25600*			1.0–3.8	--
*>1*:*25600*			7.9–26.6	35.6
*- Sero-converters DAT titres HR*	[44]			
*<1*:*1600*			reference	reference
*1600 - <25600*			6.6–9.0	10.1
*>1*:*25600*			44.5–111.0	99.2
*DAT+ve (cf DAT-ve) RR at 1y*	[48]		3.42	3.42
*Recent DAT sero-converter (cf EHC) RR at 1y*	[48]		11.5	11.5
**Immune and other markers**				
*- IFN- γ*, *NO*, *CRP*	[47,81]	Raised	Raised	
*- TNF –α*, *IL-2*, *IL-4*	[81]		Low	
*- Serum retinol*, *zinc*	[47]	Low		
**Parasite markers**				
*- parasite load (>5genome equivalent/mL)*	[46,76]		80%	

^1^: at 6mo follow up

Note: cf (compared from); CRP (C-reactive protein); DAT (Direct agglutination test); EHC (Endemic healthy controls); ELISA (Enzyme linked immunosorbent assay); HR (Hazard ratio); IFN- γ (Interferon gamma); IL-2 (Interleukin 2), IL-4 (Interleukin 4); LST (Leishmanin skin test); NO (Nitric oxides); OR (Odds ratio); PCR (Polymerase chain reaction); qPCR (quantitative polymerase chain reaction); rk39 (recombinant kinetoplast 39); RR (Relative risk); TNF –α (Tumor necrosis factor alpha)

Most infections remained asymptomatic [42]. Spontaneous resolution (sero-reverting from positive to negative status) was seen in 33–86.7% of ALI within a year [41,48,62,66]. The proportion recovering spontaneously was lower for ALI with higher antibody titres [47]. On the other hand, the proportion of ALI that progressed to symptomatic disease within one year ranged between 1.5–23% [44,48–50,58,62,68]. It was higher (18.8–69.1%) amongst seropositive contacts of VL compared to seropositive non-contacts (5–38%) [58,66,77]. Seropositive contacts were 1.64–4.82-fold more likely to progress to clinical disease compared to seropositive non-contacts of a known VL case [47,49,62]. Anti-*leishmania* antibody titres were strong predictors of progression of ALI to symptomatic VL disease. Healthy but seropositive individuals with moderate antibody titres as measured by DAT were up to 5-times, and those with high titres were 8–40-times more likely to progress to symptomatic disease than those who were seronegative [44,46]. The risk for progression to symptomatic disease was significantly greater (up to 9 and 111-fold for moderate and high titres respectively) despite the small numbers amongst sero-converters [44,48]. Raised levels of immune cytokines and chemokines such as interferon-γ, nitric oxides, C-reactive protein and lowered levels of TNF-α, interleukin-2 and interleukin-4 were other potential markers for progression of ALI to clinical disease [47,81]. Parasitaemia levels were 500-fold lower in ALI than in active VL disease; individuals with a parasitaemia >5 parasite genome / mL were at higher risk of developing clinical VL [46,76].

Xenodiagnostic studies or artificial feeding experiments were limited in scale and number. In one study, 5.3% of a total of 258 laboratory-bred *Ph argentipes* became infected when fed on active VL patients [82]. There were no studies from the Indian subcontinent that quantified the infectiveness potential of infected asymptomatic individuals. Mathematical transmission models estimated the infectiveness of an early (PCR+, DAT-) and late asymptomatic infected (PCR+, DAT+) individual to be 2.5 and 3.3% assuming that a VL patient would infect 100% of sand flies feeding on them [83,84]. The model further assumed that the relative infectiveness of an early asymptomatic infected (PCR+ but seronegative) individual was half of that of a late asymptomatic infected individual.

### PKDL–a reservoir for infection?

PKDL is hypothesized to be the reservoir for the *Leishmania* parasite and was incriminated in the resurgence of VL in West Bengal, India in the 1990s following discontinuation of insecticide spraying [85]. A total of 35 studies on PKDL including 6 reviews were identified. Except for one longitudinal study each in Nepal and India, most studies were cross-sectional surveys or based on surveillance data. There was no standard case definition for PKDL diagnosis, though operational case definitions are available from the WHO since 2010 [86]. All rk39 positive cases, with or without a past history of VL, with skin lesions suggestive of PKDL were considered probable PKDL by most studies. Confirmed PKDL required the demonstration of parasite in the skin lesion. Table 2 summarizes the findings of burden, natural history, risk factors and infectiveness of PKDL. The prevalence of confirmed PKDL ranged between 4.4–4.8 per 10,000 population in Bangladesh and India [87–89]. The incidence of PKDL has been estimated to be 0.1 per 10,000 (Table K in S1 Text). The development and natural history of PKDL showed wide variability. The proportion of PKDL without a preceding history of VL was between 3.8–28.6% [90–94]. The proportion of treated VL cases who developed PKDL within a year averaged 1–3% [95,96]. The mean period from VL treatment to development of PKDL was 1–4 years. The duration to development of PKDL did not differ by the drug used for VL treatment. The duration was slightly longer for nodular PKDL (34mo) compared to macular or papular PKDL (22.8–23.8mo). Active surveillance of a population of 24,814 individuals in Bangladesh between 2002 and 2010 identified 98 untreated PKDL patients, 48 (about 49%) of whom resolved spontaneously with a median time of 19 months [91]. The younger age group was more likely (17.1%) to develop PKDL compared to older VL cases (12.5%). They also developed PKDL earlier (27mo compared to 50mo) [87]. Family history of VL and clustering was a significant risk factor for development of PKDL [88,94]. Inadequate treatment of VL with antimonials was associated with a 11.6-fold higher risk of developing PKDL [96].

**Table 2:** PKDL as a potential reservoir of infection in the Indian subcontinent.

	Reference	Bangladesh	India	Nepal
**Burden**				
Prevalence (per 10000 pop)	[87–89]	6.28 (probable); 4.4 (confirmed)	7.8 (probable); 4.4–4.82 (confirmed)	
Incidence (per 10000 pop)	[90]	1–21		
Incident PKDL per 100 VL cases	[90,91,96]	3–9.7% (1y); 10% (2y); 17% (3y)		1.4% (2y); 2.5% (2 – 4y); 3.6% (4 – 8y);
**Natural history**				
Prop PKDL without a past h/o VL	[90–94,97–100]	9.2%	3.85–20%	28.6%
Prop of VL developing PKDL post-VL treatment	[95,96,100]		0.29–15% (5y)	1.4% (1y); 2.5% (4y); 2.9% (5y); 3.6% (8y)
VL treatment–PKDL duration	[87,88,90,92–96,98–100]	3y (2 –4y);	12mo– 3.13y (range 1mo– 20y); 23mo (post-antimonial); 29mo (post-amphotericin); 9mo (post-liposomal amphotericin); 31mo (post-miltefosine); 25mo (post-paramomycin)	23–26.9mo (range 6mo– 5y); 22.8mo (macular); 23.8mo (papular); 34mo (nodular);
Prop of PKDL by VL–PKDL duration	[93,95,97]		33.0–36.4% (1y); 68.2% (2y); 19.3–82% (<5y); 18–70.5% (>5y); Post-liposomal amphotericin: 1.2y; Post-antimonial: 2.9y (1.5–5.5y);	
Prop PKDL resolved without treatment	[91]	49%;		
Duration to PKDL resolution	[91]	19mo		
Onset–treatment time lag for PKDL	[93,98]		2y (range 1 – 12y); Onset–treatment duration varied with type of PKDL lesion;	28.4mo (macular); 26.1mo (papular); 39.5mo (nodular);
**Risk factors**				
*- Young age at VL (cf older age)*	[87,88,90,91]	OR: 1.36	Higher risk	
*- clustering*	[88]		Higher risk	
*- inadequate VL treatment*	[96]			OR: 11.68
**Infectiveness of PKDL to SF**				
*- SF infected after feeding on PKDL*	[101,102]		32–53%	

Note: cf (compared from); h/o (history of); SF (sandfly); OR (Odds ratio)

We identified two xenodiagnostic studies done on PKDL patients from the Indian subcontinent. The proportion of sandfly getting infected after feeding on PKDL patients in an experimental setting ranged from 32–53% with the highest rate of infection of the sandfly seen on the 4^th^ day post-feed [101,102]. For lack of data, transmission dynamics modelling studies assumed the infectiveness of PKDL to be either 50 or 100% in order to estimate other parameters of interest such as infectiveness of the asymptomatic stage of infection [84]. One of the model variants was structured in a way to assume that only VL and PKDL (but not ALI) contributed to transmission. With this and other assumptions of sandfly density, biting rate, life expectancy etc., the infectiveness of PKDL relative to VL was estimated by this model variant to range between 2.32–2.72 [83].

### Relapse–a potential threat to VL elimination?

A total of 20 studies relevant to relapse including eight drug trials and six cohort studies were identified from the Indian subcontinent. There was no standard case definition for relapse. Most studies required demonstration of the parasite to confirm a relapse following either a clinical cure or parasite cure at the end of treatment for VL or PKDL.

#### VL relapse

The 1 year cumulative incidence of VL relapse following VL treatment with antimonials ranged from 0.14–1.67% [103–106]. A relapse rate of up to 3.7% and up to 20% was seen following treatment with liposomal amphotericin and miltefosine respectively (Table 3). The relapse incidence was higher (8.1–67%) for HIV-co-infected individuals [103,107]. The mean duration from VL treatment to relapse ranged from 3.75 to 10 months with 68% relapsing within a year following treatment with liposomal amphotericin [29,32,108]. The risk of relapse was higher (OR 1.94–3.54) in children, and those who presented with a shorter duration of symptoms prior to VL treatment [108,109]. The risk of relapse was higher (16% at 1y, 20% at 2y and 26% at 4y) for individuals with HIV co-infection [9,21,110]. *L donovani* antigen-specific IgG1 levels were significantly elevated in treatment failure and relapsed cases compared to cured VL patients [111]. Similarly, a rise in anti-rk39 antibody titres to levels higher than pre-treatment levels following treatment indicated treatment failure and relapse [64]. Quantitative PCR could potentially define thresholds for parasite load to predict relapse but there were no studies from the Indian subcontinent [21].

**Table 3:** Relapse following VL and PKDL in the Indian subcontinent.

	Reference	Bangladesh	India	Nepal
**VL relapse**				
**Incidence of VL relapse**				
*- post-antimonial VL treatment*	[103–106]	0.6% (1y)	0.14–1.67% (1y); 67% (HIV co-infected– 1y);	
*- post-miltefosine VL treatment*	[29,31,109,112–118]		1.6–11.1% (6mo); 7.6–12.8% (1y);	6–10.8% (6mo); 12.8–20.0% (1y);
*- post liposomal amphotericin treatment*	[9,32,33,107,108,110,116]	3.6%	0–0.26% (6mo); 1.39–3.7% (1y); 8.1% (HIV co-infected - 1y); 26.5% (HIV co-infected - 2y); 17–49.1% (HIV co-infected);	
Duration to relapse distribution (post-liposomal amphotericin treatment)	[108]		15.1% (<6mo); 52.9% (6 – 12mo); 31.9% (>12mo);	
Duration to relapse	[29,32,108,110]		3.75–10.1mo; 10mo (HIV co-infected)	
**Risk factors for VL relapse following treatment with miltefosine or liposomal amphotericin**				
*- children (cf adults) OR*	[31,108,109,113]		1.94–3.54	3.19
*- male (cf female) OR*	[108,109]		1.74–2.14	2.14
*- decrease in spleen size < 0*.*5cm/day at discharge OR*	[108,109]		1.0–1.55	1.0
*- longer duration of symptoms prior to treatment OR*	[108,109]		0.62–1.0	1.0
*- Risk of relapse with HIV co-infection*	[9,21,110]		16% (1y); 20% (2y); 26% (4y); 6.4% (1y) (combination therapy)	
*- Anti-retroviral therapy for HIV co-infection after admission RR*	[110]		0.25	
**Markers for VL relapse**				
*- rk39 ELISA titres*	[64]		No correlation	
*- parasite genotype*	[31]			No difference between cured VL and relapse;
*- CD+4 count (in HIV-co-infected)*	[103,107]		<200 /cmm	
*- promastigote morphology*	[30]			Procyclic: Longer slender body; Metacyclic: shorter body; Increased metacyclogenesis;
*- macrophages*	[30]			Higher percentage of macrophages infected with parasite;
**PKDL relapse**				
Incidence of PKDL relapse following PKDL	[9,88,92,119,120]	13%	0–12.5% (post-miltefosine); 22%;	
**Risk factors for PKDL relapse**				
*- drug dosage*	[92]		No correlation;	
*- treatment duration*	[121]		43% (post-miltefosine for 3mo); 0% (post-miltefosine for 4mo);	
**Parasite resistance (drug susceptibility)**				
- promastigote survival (IC_50_)	[122]		PKDL: 11.45 (SD: 4.19); VL: 2.58 (SD: 1.58)	
*- promastigote survival (IC*_*50*_*) (VL relapse following VL)*	[31,122]		Pre-miltefosine: 1.86; Post-miltefosine cured: 2.43; Post-miltefosine relapse: 4.72;	No difference between cured VL and relapse;
*- promastigote survival (IC*_*50*_*) (PKDL relapse)*	[122]		Pre-miltefosine: 8.63; Post-miltefosine relapse: 16.13;	

Note: IC_50_ (Inhibitory concentration 50%); SD (Standard deviation); cf (compared from); OR (Odds ratio); rk39 (recombinant kinetoplast 39); ELISA (Enzyme linked immunosorbent assay)

#### PKDL relapse

The cumulative incidence of a PKDL relapse following PKDL treatment ranged from 0–12.5% [9,92]. Two of the 9 (22%) PKDL patients detected in a house to house survey were relapsed cases of PKDL [88]. There was no correlation between the miltefosine dosage used for treating PKDL and PKDL relapse [92]. However, the proportion of PKDL relapse following a 3mo PKDL treatment with miltefosine was significantly higher (43%) compared to PKDL patients treated with 4mo of miltefosine [121]. There were two case reports of recurrence of VL following PKDL possibly triggered by immunosuppression due to concurrent infection [123]. A higher proportion of macrophages infected with the parasite was reported in relapse. There was no difference in the parasite genotype but some phenotype changes and increased meta-cyclogenesis was seen [30].

Parasite resistance to drugs has also been studied as a potential cause for relapse. Overall, promastigote survival was significantly higher in intracellular amastigote cultures obtained from PKDL compared to VL patients. More importantly, promastigote survival was significantly higher in isolates from relapse cases compared to pre-treatment isolates (Table 3) [122]. In contrast, there was no difference in parasite survival between cured VL and relapse cases [31]. One transmission modelling study that modelled the effect of drug-resistant parasites on treatment failure rates, simulated different scenarios where either disease-related factors, pathogenicity (resistant parasite would cause more VL cases compared to susceptible parasite) or virulence (symptomatic VL case with resistant parasite would infect more sandfly) or transmission-related factors (asymptomatic or symptomatic host will infect more sandfly or sandfly will infect more host) were varied to predict the effect of drug resistance on treatment failure rate. The study concluded that increased transmissibility of resistant parasites (and not antimonial resistance alone) was the most likely reason for the unusually high treatment failure up to 65% seen in India between 1980–97 [27,34].

### Modelling transmission dynamics for planning and evaluation of VL elimination

We reviewed seven modelling studies that used data from the Indian subcontinent [34,80,83,84,124–127]. Transmission was modelled to quantify the levels and consequences of under-reporting, to quantify and predict the effect of different treatment or vector control strategies on VL incidence and / or prevalence and to ascertain the potential of ALI and PKDL as reservoirs of infection. All models were deterministic albeit with slightly varying compartments for the different transmission and clinical stages. The major differences in the model structure, data sources used to fit the model, assumptions, fixed and estimated parameters, scenarios simulated, main findings and limitations are summarized in Table L in S1 Text. Under-reporting can be a problem for planning and evaluation of elimination strategies. The first attempt at estimating under-reporting ratios was based on mathematical modelling that predicted a 90% under-reporting rate in 5 and 8 of the 21 endemic districts in Bihar, India in 2003 and 2005 respectively [124]. As a result, 3–5 districts were misclassified as high or low risk. Furthermore, the model predicted that the population density, health infrastructure, literacy level of the district had no effect on the extent of under-reporting which was sensitive to changes in VL endemicity levels. Community-based surveys reported an actual under-reporting of VL cases by a factor of 8.13 in 2003 [128] and 4.17 in 2005 in Bihar, India [129]. More than 85% of VL patients sought treatment from the public sector and was consistent with a downward trend in under-reporting, which was largely attributed to the free availability of VL drugs in government facilities under the elimination program [35]. Any attempt at interpreting the current reported disease trends should take into account this drastic change in underreporting ratio.

Assuming that clinical cases were responsible for the bulk of transmission, country-specific empirical data on health seeking behaviour was used to parameterize a transmission dynamics model to predict the effect of very early diagnosis (when non-specific symptoms such as fever appeared before the classical signs and symptoms of VL) and to characterize the profile of a potential diagnostic product [126]. Patients in Nepal, typically first presented with VL symptoms to the health system and had a shorter duration of onset of symptoms to diagnosis and treatment, whereas in India, patients sought care earlier at the stage of non-specific symptoms, and experienced delayed diagnosis and remained untreated for a longer duration [130]. The study shows the importance of earlier diagnosis and prompt therapy in VL elimination but also the risk that reduced transmission will expose the population to future epidemics, with waning herd immunity if vigilance is not maintained and diagnostic delays increase–a factor which might further delays the detection of an epidemic.

The models shows that the importance of novel diagnostics that can detect the infection in asymptomatic carries before they develop full VL, where high specificity is at a prime, even if sensitivity is relatively low. The reason is that the challenge of early testing with the intention of treating is to avoid false positives, especially with decreasing prevalence.

Transmission dynamics was modelled to predict the effect of treatment of VL and PKDL patients on VL elimination, simulating different scenarios of detection delays, varying duration of treatment regimens, varying rate of treatment failure and relapse [84]. The model was fitted to the KALANET data to predict a best-case treatment scenario (early case detection, shorter duration, and more efficacious treatment) to reduce VL prevalence but no effect on the prevalence of ALI. Such a scenario reduced PKDL incidence from 1 to 0.6 per 10,000 but had a minimal effect on VL incidence (the benchmark for the elimination program). The model predicted that transmission was predominantly driven by asymptomatic infected individuals and early case detection and treatment had no substantive effect on overall transmission. A variation of this model which tested the assumption of PKDL (as opposed to ALI infection) as the reservoir of infection predicted that ALI contributed to 82% of the overall transmission compared to 10% by symptomatic VL and 8% by PKDL patients [83].

Transmission dynamics was similarly modelled to predict the effect of different vector control strategies on VL elimination simulating different scenarios of optimal and sub-optimal IRS under varying endemicity levels and different assumptions of infection reservoirs. The transmission of the parasite between the host and sandfly was dependent on the infectiveness of the host or of the sandfly with a single bite, the mean biting rate and the sandfly density. The biting rate was assumed to be 0.25 / day (inverse of the feeding interval assumed to be 4 days [131]) and the latency period in the sandfly was assumed to be 5 days [132]. The elimination target could be achieved if the sandfly density was reduced from 5.27 to 1 per human, the life expectancy of the sandfly halved from 14 to 7 days, or the interval between blood feeds for the female sandfly doubled from 4 to 8 days or by a combination of any of these [84]. Entomology surveys estimated a prevalence of infected sandfly to range from 4.9–12% [64,71,133,134]. Table 4 gives details of sandfly abundance, distribution and feeding behaviour and risk factors affecting sandfly density. The effect of vector control on reducing PKDL prevalence would be delayed due to the latency between recovering from VL and developing PKDL. The same model estimated the basic reproduction number (R_0_) as 4.71 (95% CI: 4.1–5.4). The effective reproduction number (R_e_) was reduced non-linearly by IRS and LLINs and linearly by environmental management for vector control [125]. The model predicted that VL would be eliminated if the sandfly density was reduced by 67% (95% CI: 60–72%) by killing the sandfly with IRS or LLINs or if the sandfly density was reduced by 79% (95% CI: 75–82%) by reducing their breeding sites with environmental management for vector control or by a combination of these. Compared with these model predictions, the actual reduction in sandfly density of 24.9–43.7% with LLIN [135,136] and 42% with environmental management for vector control [136] seen in intervention trials (Table 4), would not be sufficient to reduce transmission to achieve the VL elimination target. However, the sandfly reduction of 72.4% seen in the intervention trial [136] with IRS would be adequate to reduce the transmission level (R_e_<1) to achieve elimination. A more recent model designed to test different scenarios of optimal and sub-optimal application of IRS (sandfly density reduced by 63% and 31.5% respectively) in varying endemicity settings predicted that optimal use of IRS reduced the VL incidence by 25% and 50% at 1y and 2y respectively at all endemicity levels [83]. VL incidence continued to decline as the burden of ALI became less. However, the decline in VL incidence was slower if PKDL (not ALI) was assumed to be the main reservoir of infection. The model predicted VL elimination with 4 – 6y of optimal IRS or 10y of sub-optimal IRS and only in low endemic (VL incidence < 5 / 10,000) setting whereas VL was not eliminated even with 12y of optimal IRS if PKDL were assumed to be the main reservoir of infection. A longer period to development of PKDL, a longer PKDL duration increased the transmission pressure to slow down the decline in VL incidence. Model predictions of VL elimination by IRS depended on the assumptions about the main reservoir for infection (ALI or PKDL) and were sensitive to other model assumptions such as the proportion of ALI progressing to symptomatic disease and the proportion of VL developing PKDL. However these predictions were robust to assumptions of infectiveness of early asymptomatic relative to that of late asymptomatic stage.

**Table 4:** Sandfly abundance, infectiveness, risk factors and effects of vector control strategies.

	Reference	Bangladesh	India	Nepal
**SF abundance**				
SF distribution	[137,138]		Vegetation (30.6%), mixed dwelling (26.7%), cattle shed (18.6%), human dwelling (12.1%), chicken coop (12%); Cattle shed, mixed dwelling (77%);	
SF density	[139–141]	Human dwelling: 10.22 SF/MH; Mixed dwelling: 17.09 SF/MH	Human dwelling: 25 SF/MH Cattle shed: 100 SF/MH; Peak: 5.60 SF/MH	4.4 female SF/MH;
SF saliva antibody titres in human	[45,142]		43.5–63.2% Positive correlation with female SF density;	43.5–63.2%
Prevalence of infected SF	[71,133,134,143]		Microscopy: 0.1%; PCR+: 4.9–17.37%; PCR+: 1.5% (annual), 2.84% (winter), 1.04% (summer), 0.85% (monsoon);	PCR+: 12%
Prop SF infected after feeding on infected host	[82,143] [144]		2.43–5.33%; 100%	
SF feeding preference	[145]		Cattle: 68%; human: 17.9%; birds: 4%;	
**Factors affecting SF density**				
*- seasonality*	[134,137,140,146–150]	Peak: Mar; Blood-fed SF peak: May;	5.60–13.0 SF/MH (peak season); 2.13–8 SF/MH (lean season); 10.09–11.14 SF/trap (Sept–Oct); 0.28–0.37 SF/trap (Jan–Feb);	Peak: Mar, Sept;
*- indoor temperature*	[148,151]		29–32°C (peak season); 20–24°C (lean season);	
*- relative humidity*	[151]		Predicts SF abundance	
*- soil pH*, *soil moisture*	[150,151]		Alluvial soil;	
*- vegetation*	[146,151]		Inversely correlation	
*- land use*, *land cover suitability for SF habitat*	[151,152]		High suitability: water bodies, sandy area, moist fallow area, weeds, grassland, near water body, marshy land, dry fallow, settlement; Low suitability: plantation;	
*- VL prevalence*	[134,146]		No correlation	
**Effect of SF control**				
*- IRS effect and duration*	[5,136,147,153–155]	Rebound at 11mo; 94% reduction at 6mo;	Rebound at 3mo; 124% reduction at 6mo; Human dwelling: 4.5 SF/MH at 1mo; Mixed dwelling: 5 SF/MH at 1mo; Cattle shed: 6 SF/MH at 1mo; No SF after 2^nd^ IRS at 1.5mo interval;	Reduced from 11 to 0.6 SF/trap; 52–53% reduction at 6mo;
*- LLIN effect and duration*	[136,141,147,154]	60% lower at 11mo; 68% reduction at 6mo;	298%^1^ reduction at 6mo;	Reduced from 7.9 to 0.9 SF/trap; 16–22% (NS) reduction at 6mo;
*- EVM effect and duration*	[136,154,156]	9% reduction at 6mo;	108%^1^ reduction at 6mo;	Reduced from 8.2 to 2.6 SF/trap; 4–51% reduction at 6mo;

^1^ Larger than 100% as the SF density decreased in intervention group but increased in control group at 6mo

Note: SF (Sandfly); SF/MH (Sandfly per man-hour); PCR+ (Polymerase Chain Reaction positive); NS (not significant); EVM (environmental management for vector control); IRS (Indoor residual spraying); LLIN (Long lasting insecticide nets)

## Discussion

We identified eight systematic reviews including two Cochrane reviews–five reviews were on diagnostic tests and biomarkers, and one review each on treatment options, risk factors, and vector control. This is the first systematic review of transmission dynamics from the perspective of VL elimination in the Indian subcontinent.

The burden of ALI relative to active VL disease was high in the Indian subcontinent. The thresholds for serodiagnosis were originally defined for active VL disease and need to be validated for diagnosis of ALI. Studies of ALI were hampered by lack of a reference standard and appropriate biomarker which also hampers the evaluation of any new assay. However, any assay used could at least be evaluated to be 100% specific in a sufficiently large group of non-endemic controls. Molecular techniques such as PCR and q-PCR are now increasingly used to diagnose early ALI with parasitaemia levels as low as less than one parasite genome per mL of blood. At such low levels, it would be important to know whether the parasite DNA detected by PCR was from a metabolically-active parasite and not just a residual DNA from a dead parasite. The parasite load in ALI was reported to be 500-fold lower than that in active VL disease [76]. A study from Ethiopia that modelled the *L donovani* parasite load in the blood of asymptomatic and symptomatic individuals to estimate their infectiveness to the sandfly species *Ph orientalis* predicted that 3.2% of the most heavily-infected individuals (parasitaemia levels greater than 500 parasites genomes / mL) were responsible for 62% (95% CI: 53–79%) of the infected sandfly population [157]. Based on the parasite load threshold of <5 per mL for ALI [76], this implied that symptomatic infection was the predominant driver for transmission and by implication treatment of symptomatic VL cases would be most effective to reduce transmission. Nevertheless, AVL may continue to act as a hidden reservoir of infection which will build up to eventually lead to another epidemic outbreak in the presence of vector abundance. The infectiveness of HIV–*Leishmania* co-infected patients maybe higher due to higher parasitaemia levels [158]. Laboratory studies from Ethiopia suggest a *L donovani* parasite load of 20,000 per mL of blood in the host is required to infect the sandfly species *Ph orientalis* [159]. Infectiousness was seen to be correlated with parasite loads in dogs exposed to natural *L infantum* infection [160]. The threshold for parasitaemia at which a sandfly that ingests less than 1 μL of blood during a feed on an infected host can get infected needs to be defined through xenodiagnostic studies or artificial feeding experiments. Studies using different modelling approaches, assumptions and data sets gave contradictory results–one study predicted that ALI contributed to 82% of the overall transmission whereas another study predicted that 3.2% of infected human VL cases with the highest parasitemia were responsible for 62% of all the infections in sandfly [83,157]. It seems likely that only those persons with very high parasitaemia would serve as effective reservoirs infecting the sandfly [161]. Nevertheless, even if the transmission potential for ALI is relatively low compared to active VL or PKDL when prevalence is high, their potential should not be underestimated as they represent a large reservoir of infection [162]. There is now evidence from outbreak investigations in new eco-niches that suggest that local transmission can be maintained in isolated communities in the absence of VL or PKDL cases [8,163]. Further research is needed to define markers and thresholds for defining ALI, for infectiveness to the sandfly and to predict the potential for progression of ALI to symptomatic VL disease.

PKDL is a poorly understood sequelae of VL. Most of the evidence on the natural history of PKDL came from epidemiological and clinical studies. Given the long and variable duration to the development of PKDL, it is logistically difficult to conduct longitudinal studies. Research on PKDL is also hampered by a lack of a validated definition, appropriate biomarkers, and standardized definition for treatment end-point and cure. Some case definitions biased the selection of PKDL cases to only those with a past history of VL treatment. Nevertheless, the burden of PKDL in the Indian subcontinent was considerable. It was suggested that as few as 0.5% of PKDL cases during a VL epidemic can potentially sustain transmission to make VL endemic [164]. Differences in methodology to detect PKDL cases (active case detection in Bangladesh, passive surveillance based on patients with the more severe nodular form of PKDL reporting to health facilities in India), differences in treatment practices for VL (antimonial treatment for 3 weeks in Bangladesh compared to 4 weeks in India and Nepal), and treatment compliance may partly explain the higher PKDL burden seen in Bangladesh compared to India and Nepal. Further research is needed to understand differences in pathogenesis of PKDL within the Indian subcontinent. It has been presumed that PKDL is a potential reservoir for infection that may trigger a recrudescence of the disease post-elimination as was seen in the 1990s in India. On the other hand, PKDL has not been identified as the source of outbreaks in previously non-endemic areas in the last two decades. There are many unresolved issues for PKDL. The continental differences in the pathogenesis, immune response and transmission of PKDL prevent extrapolation of findings from Africa to the Indian subcontinent [165,166]. The potential role of qPCR in predicting the development of PKDL needs to be studied. The parasitaemia threshold for infectiveness of PKDL needs to be defined as parasites are more likely to be in the skin lesions relative to their presence in blood. Moreover, the infectiveness thresholds are likely to differ for the nodular form of PKDL compared to the maculo-papular form which is far more frequent in the Indian subcontinent. Experimental studies have shown that 32–53% of laboratory bred sandfly were infected after feeding on PKDL patients [101,102]. Modelling studies have assumed the infectiveness of PKDL to be the same or half of that of an active VL case. Further research is needed to define markers and thresholds for the development of PKDL and their infectiveness to the sandfly to better understand the role of PKDL as a potential reservoir especially in the maintenance phase of VL elimination.

The demonstration of parasite in the blood of domestic animals and the significant spatial clustering of PCR-positive animals and humans suggests domestic animals as an alternate reservoir for an infection thought to be only anthroponotic in transmission on the Indian subcontinent [53]. In mixed dwellings where cattle sheds are attached to the house, 66% of the blood meals of the sandfly was of bovine origin whereas 19% of the sandfly fed on human [145]. In contrast, 81–92% of the blood meals were of bovine origin from cattle sheds compared to 49–100% blood meals of human origin in human dwellings [145,167,168]. Domestic animals could increase transmission pressure by virtue of being an untreated reservoir for the parasite. Proximity to domestic animals may also increase sandfly density, and as a result transmission, due to increased availability of blood meals for the sandfly as well as organic manure for breeding of larvae and resting. On the other hand, proximity to domestic animals may lower transmission by altering sandfly feeding behaviour and lessen the human exposure to sandfly [36,47,51]. Even in the scenario where domestic animals are just reservoirs for infection without zoonotic transmission, they could be a potential threat to VL elimination unless they alter sandfly feeding behaviour and lower human exposure to sandfly bite. Further research is needed to understand the potential of domestic animals as a reservoir for infection and its complex effect on VL transmission.

Though the burden of relapse is relatively low following treatment with miltefosine or liposomal amphotericin, it is important in the context of transmission dynamics. First, relapse contributes to the overall infective pool of parasite in the host that is available for transmission to the sandfly. Second, in HIV positive individuals who are not on antiretroviral therapy, VL relapse increases the risk of transmission because of the suppressed immunity and higher parasite load and unresponsiveness to drug treatment [21]. Third, the possibility of parasite resistance to anti-*Leishmania* drugs seen in patients with HIV co-infection experiencing relapse may be an important reservoir for drug-resistant parasite by being parasitaemic for longer periods or for their increased risk to develop PKDL [169].

Treatment outcome measures should be standardised; the case definition of relapse varied among studies. Initial cure was assessed on clinical resolution and / or parasitological cure at intervals ranging from 1–2 months post-treatment [112,113]. Final definitive cure was assessed clinically at intervals ranging from 6–12 months post-treatment [32,108]. Most studies were hospital-based and could not differentiate between reinfection, reactivation and relapse [31]. They were not powered or designed to follow up patients post-treatment long enough to estimate relapse rates. Though the burden of VL–HIV co-infection is relatively lower on the Indian subcontinent than in Africa, the importance for monitoring for relapse and emergence of drug-resistant parasites cannot be overemphasized.

The interest in modelling VL transmission to inform VL elimination program strategies is recent and comes primarily from two groups of modellers from the Neglected Tropical Diseases Modelling Consortium. The proportion and progression of the various infective stages in transmission cycle, the persistence of antibodies and acquired immunity following infection, the host and vector infectiveness profile need to be based on large well-designed and powered longitudinal field trials so as to better parameterize the model [20]. Models on vector control measures do not consider the duration of the post-intervention effects relative to the duration of infectiveness by individuals (whether VL, ALI or PKDL) if not ‘removed’. Current models need to be further refined to allow for stochastic variation, factor-in the effect of temporal and spatial clustering of cases, and the effect of acquired immunity on transmission dynamics. Further research is needed on vector bionomics, the potential role of an alternate animal reservoir for infection and its complex effect on transmission dynamics.

Our review, though systematic, was subject to several methodological and substantive limitations. First, we restricted our review to literature in the English language. However, we were aware that the majority of the research work on VL in the Indian subcontinent has come from a relatively small community of researchers and institutes historically and as we contacted the key persons in this network for additional information, it is unlikely that we may have missed out on any substantive evidence. Second, as we restricted the scope of our systematic review to the Indian subcontinent, we may have missed out on some lessons learnt from VL studies in other continents, lessons that could be partly or wholly extrapolated to the Indian subcontinent. Third, we did not do a systematic review of the sandfly biology component of the transmission cycle. We hope this component will be covered in a separate systematic review. Fourth, we did not cover other reported modes of transmission such as through blood transfusion [170], organ transplant [171,172], laboratory accidents [173] and the occasional reports of mother to child transmission [174]. These transmission routes are very rare. The mechanical transmission of the *Leishmania* parasite through syringes used by intravenous drug users is probably more relevant [175]. Though currently not yet problematic, the rising number of HIV–*Leishmania* co-infections is gaining importance in the region as also in the risk group of IV drug users is present in more urban communities.

As progress towards VL elimination gains momentum, there are many ongoing and planned research efforts aimed at improving treatment and vector control strategies [9]. Research is ongoing for improved diagnostics and identifying biomarkers for ALI. A longitudinal study in Bihar, India is following a cohort of asymptomatic cases in households with and without current or past VL case using q-PCR to assess the parasite load thresholds for progression of ALI to VL disease. Another longitudinal study of VL and PKDL patients in Bangladesh is ongoing to estimate relapse rates and risk factors. The role of treating livestock to interrupt transmission is being investigated. A transmission dynamics study and evaluation of the impact of disease elimination is being planned in endemic villages of Bihar in India. Research is now focused on investigating new outbreaks in hitherto non-endemic areas to understand the source and risk factors for the indigenous transmission dynamics. Modelling groups are working towards refining their models to simulate the effects of scenarios that could be a potential threat to elimination efforts. All these and other planned and ongoing research are urgently needed to help national programs to achieve and maintain elimination of VL in the Indian subcontinent.

### Conclusion

The burden of ALI is considerable. Longitudinal studies are necessary to identify biomarkers for infectiveness and for progression of ALI to symptomatic VL disease. More research is needed on the immune response to VL and PKDL to identify biomarkers for development of PKDL. Xenodiagnostic studies are necessary to quantify the infectiveness of ALI and PKDL to sandfly relative to symptomatic VL, and their contribution to overall transmission. Even though domestic animals are seen to be infected, there is no evidence of their role in anthroponotic transmission in the Indian subcontinent. Relapse rates need to be monitored for their potential to contribute to transmission and for the emergence of drug-resistant parasites in the context of HIV co-infection. Availability of better data from large well-designed longitudinal studies for modelling would contribute to a better understanding of the impact of treatment and vector control strategies and potential threats to VL elimination in the Indian subcontinent.

## Supporting Information

S1 Text Supplementary information containing unpublished information (Tables A, B, C, J and K), PRISMA statement (Table D), Strategies and keywords used for literature search (Table E), Potential risk of bias in studies (Tables F to I), and Modelling transmission of Leishmania donovani infection in the Indian subcontinent (Table L).
(DOCX)
