# Supplementary material for: Transmission Dynamics of Visceral Leishmaniasis in the Indian Subcontinent – A Systematic Literature Review
Source: PLoS Negl Trop Dis. 2016 Aug 4;10(8):e0004896. doi: 10.1371/journal.pntd.0004896 (PMC4973965; doi:10.1371/journal.pntd.0004896)
Supplement: S1 Text — Supplementary information containing unpublished information (Tables A, B, C, J and K), PRISMA statement (Table D), Strategies and keywords used for literature search (Table E), Potential risk of bias in studies (Tables F to I), and Modelling transmission of Leishmania donovani infection in the Indian subcontinent (Table L). (DOCX) [file pntd.0004896.s001.docx]

**Table A: Data presented at the TDR-WHO expert meeting on VL transmission dynamics and continued sources of infection during the post-VL elimination phase (maintenance phase) in the Indian sub-continent, Kathmandu, Nepal (30 March – 1 April 2016)**

**** **
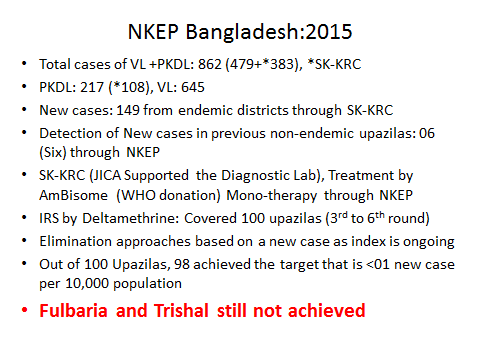
**

**Table B: Data presented at the TDR-WHO expert meeting on VL transmission dynamics and continued sources of infection during the post-VL elimination phase (maintenance phase) in the Indian sub-continent, Kathmandu, Nepal (30 March – 1 April 2016)**

**
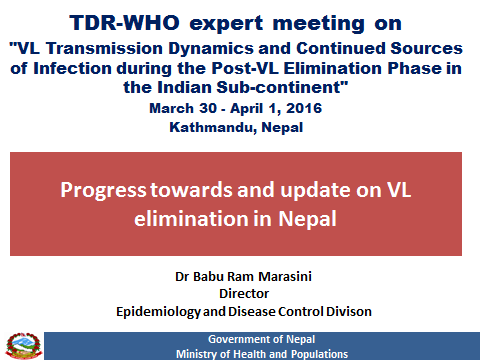
** **
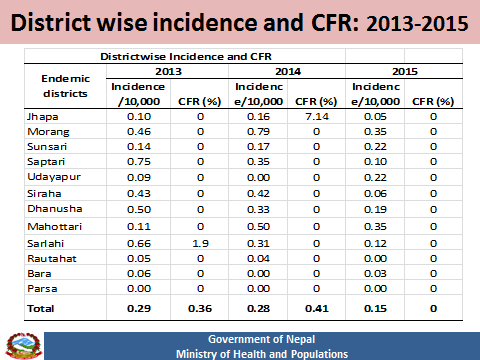
**

**Table C: Data presented at the TDR-WHO expert meeting on VL transmission dynamics and continued sources of infection during the post-VL elimination phase (maintenance phase) in the Indian sub-continent, Kathmandu, Nepal (30 March – 1 April 2016)**

**
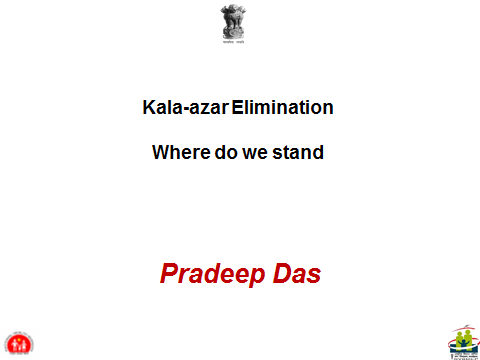
** **
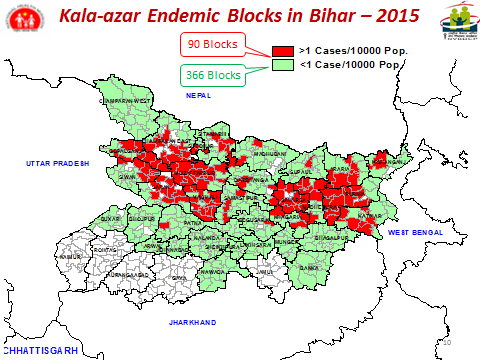
**

**Table D: PRISMA 2009 checklist**

| **Section/topic** | **#** | **Checklist item** | **Reported on page #** |
| --- | --- | --- | --- |
| **TITLE** | | |  |
| Title | 1 | Identify the report as a systematic review, meta-analysis, or both. | 1 |
| **ABSTRACT** | | |  |
| Structured summary | 2 | Provide a structured summary including, as applicable: background; objectives; data sources; study eligibility criteria, participants, and interventions; study appraisal and synthesis methods; results; limitations; conclusions and implications of key findings; systematic review registration number. | 2 |
| **INTRODUCTION** | | |  |
| Rationale | 3 | Describe the rationale for the review in the context of what is already known. | 5 |
| Objectives | 4 | Provide an explicit statement of questions being addressed with reference to participants, interventions, comparisons, outcomes, and study design (PICOS). | 6 |
| **METHODS** | | |  |
| Protocol and registration | 5 | Indicate if a review protocol exists, if and where it can be accessed (e.g., Web address), and, if available, provide registration information including registration number. |  |
| Eligibility criteria | 6 | Specify study characteristics (e.g., PICOS, length of follow-up) and report characteristics (e.g., years considered, language, publication status) used as criteria for eligibility, giving rationale. | 8 |
| Information sources | 7 | Describe all information sources (e.g., databases with dates of coverage, contact with study authors to identify additional studies) in the search and date last searched. | 7 |
| Search | 8 | Present full electronic search strategy for at least one database, including any limits used, such that it could be repeated. | Table S2 |
| Study selection | 9 | State the process for selecting studies (i.e., screening, eligibility, included in systematic review, and, if applicable, included in the meta-analysis). | 7 |
| Data collection process | 10 | Describe method of data extraction from reports (e.g., piloted forms, independently, in duplicate) and any processes for obtaining and confirming data from investigators. | 9 |
| Data items | 11 | List and define all variables for which data were sought (e.g., PICOS, funding sources) and any assumptions and simplifications made. | 9 |
| Risk of bias in individual studies | 12 | Describe methods used for assessing risk of bias of individual studies (including specification of whether this was done at the study or outcome level), and how this information is to be used in any data synthesis. | 9 |
| Summary measures | 13 | State the principal summary measures (e.g., risk ratio, difference in means). | 9 |
| Synthesis of results | 14 | Describe the methods of handling data and combining results of studies, if done, including measures of consistency (e.g., I^2^) for each meta-analysis. | Not applicable |
| Risk of bias across studies | 15 | Specify any assessment of risk of bias that may affect the cumulative evidence (e.g., publication bias, selective reporting within studies). | 9 |
| Additional analyses | 16 | Describe methods of additional analyses (e.g., sensitivity or subgroup analyses, meta-regression), if done, indicating which were pre-specified. | Not applicable |
| **RESULTS** | | |  |
| Study selection | 17 | Give numbers of studies screened, assessed for eligibility, and included in the review, with reasons for exclusions at each stage, ideally with a flow diagram. | 9 |
| Study characteristics | 18 | For each study, present characteristics for which data were extracted (e.g., study size, PICOS, follow-up period) and provide the citations. | 9 |
| Risk of bias within studies | 19 | Present data on risk of bias of each study and, if available, any outcome level assessment (see item 12). | Table S3 – S6 |
| Results of individual studies | 20 | For all outcomes considered (benefits or harms), present, for each study: (a) simple summary data for each intervention group (b) effect estimates and confidence intervals, ideally with a forest plot. | Not applicable |
| Synthesis of results | 21 | Present results of each meta-analysis done, including confidence intervals and measures of consistency. | Not applicable |
| Risk of bias across studies | 22 | Present results of any assessment of risk of bias across studies (see Item 15). | Not applicable |
| Additional analysis | 23 | Give results of additional analyses, if done (e.g., sensitivity or subgroup analyses, meta-regression [see Item 16]). | Not applicable |
| **DISCUSSION** | | |  |
| Summary of evidence | 24 | Summarize the main findings including the strength of evidence for each main outcome; consider their relevance to key groups (e.g., healthcare providers, users, and policy makers). | 29 |
| Limitations | 25 | Discuss limitations at study and outcome level (e.g., risk of bias), and at review-level (e.g., incomplete retrieval of identified research, reporting bias). | 32 |
| Conclusions | 26 | Provide a general interpretation of the results in the context of other evidence, and implications for future research. | 34 |
| **FUNDING** | | |  |
| Funding | 27 | Describe sources of funding for the systematic review and other support (e.g., supply of data); role of funders for the systematic review. | 34 |

Table E: Strategies and keywords used for literature search

| **No.** | **Query** | **Yield** |
| --- | --- | --- |
| 1 | ("Leishmaniasis, Visceral"[MeSH] OR "kala azar"[tiab]) AND "humans"[MeSH] | 5395 |
| 2 | (("Leishmaniasis, Visceral"[MeSH] OR "kala azar"[tiab]) AND "humans"[MeSH]) AND "Disease Transmission, Infectious"[MeSH] | 468 |
| 3 | ((("Leishmaniasis, Visceral"[MeSH] OR "kala azar"[tiab]) AND "humans"[MeSH]) AND "Disease Transmission, Infectious"[MeSH]) AND ("Bangladesh"[MeSH] OR "India"[MeSH] OR "Nepal"[MeSH]) | 63 |
| 4 | (("Leishmaniasis, Visceral"[MeSH] OR "kala azar"[tiab]) AND "humans"[MeSH]) AND (“Recurrence”[MeSH] OR “relapse”[MeSH]) | 146 |
| 5 | (("Leishmaniasis, Visceral"[MeSH] OR "kala azar"[tiab]) AND "humans"[MeSH]) AND (“Recurrence”[MeSH] OR “relapse”[MeSH]) AND ("Bangladesh"[MeSH] OR "India"[MeSH] OR "Nepal"[MeSH]) | 16 |
| 6 | (“Leishmaniasis, Visceral”[MeSH] OR “kala azar” [tiab]) AND “humans”[MeSH] AND “Risk Factors” [MeSH] | 184 |
| 7 | (“Leishmaniasis, Visceral”[MeSH] OR “kala azar” [tiab]) AND “humans”[MeSH] AND “Risk Factors” [MeSH] AND ("Bangladesh"[MeSH] OR "India"[MeSH] OR "Nepal"[MeSH]) | 29 |
| 8 | (“Leishmaniasis, Visceral”[MeSH] OR “kala azar” [tiab]) AND “humans”[MeSH] AND “Asymptomatic infections” [MeSH] | 19 |
| 9 | (“Leishmaniasis, Visceral”[MeSH] OR “kala azar” [tiab]) AND “humans”[MeSH] AND “Asymptomatic infections” [MeSH] AND ("Bangladesh"[MeSH] OR "India"[MeSH] OR "Nepal"[MeSH]) | 6 |
| 10 | ("Leishmaniasis, Visceral"[MeSH] OR "kala azar"[tiab]) AND "humans"[MeSH] AND "Basic reproduction number"[MeSH] | 0 |
| 11 | ("Leishmaniasis, Visceral"[MeSH] OR "kala azar"[tiab]) AND "humans"[MeSH] AND "force of infection"[tw] | 2 |
| 12 | ("Leishmaniasis, Visceral"[MeSH] OR "kala azar"[tiab]) AND "humans"[MeSH] AND "force of infection"[tw] AND ("Bangladesh"[MeSH] OR "India"[MeSH] OR "Nepal"[MeSH]) | 0 |
| 13 | ("Leishmaniasis, Visceral"[MeSH] OR "kala azar"[tiab]) AND "humans"[MeSH] AND "transmission risk"[tw] | 6 |
| 14 | ("Leishmaniasis, Visceral"[MeSH] OR "kala azar"[tiab]) AND "humans"[MeSH] AND "transmission risk"[tw] AND ("Bangladesh"[MeSH] OR "India"[MeSH] OR "Nepal"[MeSH]) | 1 |
| 15 | ("Leishmaniasis, Visceral"[MeSH] OR "kala azar"[tiab]) AND "humans"[MeSH] AND "Contact tracing"[MeSH] | 1 |
| 16 | ("Leishmaniasis, Visceral"[MeSH] OR "kala azar"[tiab]) AND "humans"[MeSH] AND "Contact tracing"[MeSH] AND ("Bangladesh"[MeSH] OR "India"[MeSH] OR "Nepal"[MeSH]) | 0 |
| 17 | ("Leishmaniasis, Visceral"[MeSH] OR "kala azar"[tiab]) AND "humans"[MeSH] AND "family"[tw] AND ("Bangladesh"[MeSH] OR "India"[MeSH] OR "Nepal"[MeSH]) | 22 |
| 18 | ("Leishmaniasis, Visceral"[MeSH] OR "kala azar"[tiab]) AND "humans"[MeSH] AND "household"[tw] AND ("Bangladesh"[MeSH] OR "India"[MeSH] OR "Nepal"[MeSH]) | 30 |
| 19 | ("Leishmaniasis, Visceral"[MeSH] OR "kala azar"[tiab]) AND "humans"[MeSH] AND "clustering"[tw] AND ("Bangladesh"[MeSH] OR "India"[MeSH] OR "Nepal"[MeSH]) | 7 |
| 20 | ("Leishmaniasis, Visceral"[MeSH] OR "kala azar"[tiab]) AND "humans"[MeSH] AND "disease progression"[tw] AND ("Bangladesh"[MeSH] OR "India"[MeSH] OR "Nepal"[MeSH]) | 9 |
| 21 | ("Leishmaniasis, Visceral"[MeSH] OR "kala azar"[tiab]) AND "humans"[MeSH] AND "Disease outbreaks"[MeSH] AND ("Bangladesh"[MeSH] OR "India"[MeSH] OR "Nepal"[MeSH]) | 37 |
| 22 | ((("Leishmaniasis, Visceral"[MeSH] OR "kala azar"[tiab]) AND "humans"[MeSH]) AND "Surveillance"[tiab]) AND ("Bangladesh"[MeSH] OR "India"[MeSH] OR "Nepal"[MeSH]) | 23 |
| 23 | ((("Leishmaniasis, Visceral"[MeSH] OR "kala azar"[tiab]) AND "humans"[MeSH]) AND "Epidemiology"[tiab]) AND ("Bangladesh"[MeSH] OR "India"[MeSH] OR "Nepal"[MeSH]) | 20 |
| 23 | ((("Leishmaniasis, Visceral"[MeSH] OR "kala azar"[tiab]) AND "humans"[MeSH]) AND ("Incidence"[tiab] OR "Prevalence"[tiab])) AND ("Bangladesh"[MeSH] OR "India"[MeSH] OR "Nepal"[MeSH]) | 94 |

Note: [tiab] – title abstract; [tw] – text word; [MeSH] – medical subject heading;

Table F: Potential risk of bias (shaded in grey) in cohort studies. Risk of bias assessed using Newcastle-Ottawa Scale

| **Potential risk of bias in** | | | | | | | | | |
| --- | --- | --- | --- | --- | --- | --- | --- | --- | --- |
| **Author**  **(Year)** | **Refer**  **ence** | **Selection of exposed cohort** | **Selection of non-exposed cohort** | **Exposure ascertainment** | **Outcome not present at start of study** | **Comparability of exposed and non-exposed cohort** | **Outcome ascertainment** | **Follow up not long enough** | **Loss to follow up** |
| Bern (2007) | [[1](#_ENREF_1)] | No | No | No | No | No | No | No | Yes |
| Bimal (2005) | [[2](#_ENREF_2)] | No | No | No | No | Yes | No | No | Yes |
| Burza (2014) | [[3-6](#_ENREF_3)] | No | No | No | No | No | No | No | No |
| Gidwani (2009, 2011)  Hasker (2013)  Singh (2010)  Sudarshan (2014) | [[7-12](#_ENREF_7)] | No | No | No | No | Yes | No | No | Yes |
| Huda (2013) | [[13](#_ENREF_13)] | Yes | Yes | No | No | Yes | No | Yes | Yes |
| Islam (2012) | [[14](#_ENREF_14)] | No | No | No | No | No | No | No | Yes |
| Kumar (2001) | [[15](#_ENREF_15)] | Yes | Yes | No | No | Yes | No | Yes | Yes |
| Maurya (2005) | [[16](#_ENREF_16)] | Yes | Yes | No | No | Yes | No | Yes | Yes |
| Ostyn (2014)  Rijal (2013) | [[17](#_ENREF_17), [18](#_ENREF_18)] | No | No | No | No | No | No | No | No |
| Singh (2002) | [[19](#_ENREF_19)] | No | No | No | No | Yes | No | No | Yes |
| Sinha (2010) | [[20](#_ENREF_20)] | Yes | Yes | No | No | Yes | No | Yes | Yes |
| Sinha (2011)  Burza (2014) | [[21](#_ENREF_21), [22](#_ENREF_22)] | No | No | No | No | Yes | No | No | Yes |
| Topno (2010) | [[23](#_ENREF_23)] | No | No | No | No | No | No | No | Yes |
| Uranw (2011) | [[24](#_ENREF_24)] | No | No | No | No | Yes | No | No | Yes |
| Vallur (2014) | [[25](#_ENREF_25)] | Yes | Yes | No | No | Yes | No | No | Yes |

Table G: Potential risk of bias (shaded in grey) in case control studies – Risk of bias assessed using Newcastle-Ottawa Scale

| **Potential risk of bias in** | | | | | | | | | |
| --- | --- | --- | --- | --- | --- | --- | --- | --- | --- |
| **Author**  **(Year)** | **Refer**  **ence** | **Case selection** | **Case representativeness** | **Control selection** | **Control representativeness** | **Comparability of case and control** | **Exposure ascertainment** | **Exposure ascertainment different for control** | **Non-response rate** |
| Ansari (2007) | [[26](#_ENREF_26)] | No | Yes | No | Yes | Yes | No | No | Yes |
| Ostyn (2015) | [[27](#_ENREF_27)] | No | No | No | No | No | No | No | Yes |

Table H: Potential risk of bias in experimental studies – Risk of bias assessed using Cochrance risk of bias assessment tool. High risk of potential bias or lack of information to assess risk of bias is shaded in grey.

| **Potential risk of bias in** | | | | | | | | |
| --- | --- | --- | --- | --- | --- | --- | --- | --- |
| **Author**  **(Year)** | **Refer**  **ence** | **Allocation sequence generation** | **Allocation concealment** | **Blinding of participants** | **Blinding of investigator** | **Blinding of outcome assessor** | **Completeness of outcome data** | **Outcome reporting** |
| Bhattarai (2009, 2010)  Gidwani (2011a)  Hasker (2014)  Khanal (2010)  Ostyn (2011)  Picado (2010, 2014)  Rijal (2010)  Singh (2012)  Srivastava (2013) | [[28-37](#_ENREF_28)] | Low | Unclear | Unclear | Unclear | Low | Low | Low |
| Joshi (2009) | [[38](#_ENREF_38)] | Unclear | Unclear | Low | High | Unclear | Low | Low |
| Singh (2006) | [[39](#_ENREF_39)] | Low | Unclear | Unclear | Unclear | Unclear | Low | Low |

Table I: Potential risk of bias in cross-sectional studies – Risk of bias assessed using Newcastle Ottawa scale adapted for cross-sectional studies. High risk of potential bias or lack of information to assess risk of bias is shaded in grey.

| **Potential risk of bias in** | | | | | | | | |
| --- | --- | --- | --- | --- | --- | --- | --- | --- |
| **Author**  **(Year)** | **Refer**  **ence** | **Sample representativeness** | **Sample size justified** | **Comparability of non-respondents** | **Exposure ascertainment** | **Comparability of different outcome groups** | **Outcome assessment** | **Statistical appropriateness** |
| Bern (2006) | [[40](#_ENREF_40)] | No | Yes | Yes | No | No | No | No |
| Das (2012) | [[41](#_ENREF_41)] | No | Yes | Yes | No | No | No | No |
| Garg (2001) | [[42](#_ENREF_42)] | No | Yes | No | No | Yes | No | No |
| Gurubacharya (2006) | [[43](#_ENREF_43)] | Yes | Yes | Yes | No | Yes | No | Yes |
| Koirala (2004) | [[44](#_ENREF_44)] | Yes | Yes | Yes | No | Yes | No | No |
| Kumar (2006) | [[45](#_ENREF_45)] | No | Yes | Yes | No | Yes | No | No |
| Mondal (2010) | [[46](#_ENREF_46)] | No | Yes | Yes | No | Yes | No | No |
| Nandy (1987) | [[47](#_ENREF_47)] | No | Yes | Yes | No | Yes | No | Yes |
| Rahman (2010) | [[48](#_ENREF_48)] | No | Yes | Yes | No | Yes | No | No |
| Rai (1989) | [[49](#_ENREF_49)] | No | Yes | Yes | No | Yes | No | Yes |
| Saha (2009) | [[50](#_ENREF_50)] | No | Yes | Yes | No | Yes | No | No |
| Saran (1992) | [[51](#_ENREF_51)] | Yes | Yes | Yes | No | Yes | No | Yes |
| Schenkel (2006) | [[52](#_ENREF_52)] | No | No | Yes | No | No | No | No |
| Singh (2013) | [[53](#_ENREF_53)] | No | Yes | Yes | No | Yes | No | Yes |

**Table J:** Ratio of incident asymptomatic infection to incident clinical disease in prospective population studies conducted in the Indian subcontinent between 2002 and 2015 ^1^

| Country | Study site | Incidence Rate VL (per 1000 person-years) | Incidence Rate ALI  (per 1000 person-years) | Ratio ALI to VL | Reference |
| --- | --- | --- | --- | --- | --- |
| India | TMRC old | 1 | 14 | 17 : 1 | Hasker et al 2014 |
| Nepal | KALANET | 1 | 16 | 13:1 | Picado et al 2010; Ostyn *et al*. 2011 |
| India | KALANET | 4 | 36 | 9:1 | Picado et al 2010; Ostyn *et al*. 2011 |
| India | TMRC new | 11 | 66 | 6:1 | Hasker et al 2014 |
| Bangladesh | Fulbaria | 16 | 63 | 4:1 | Bern *et al.* 2007 |

^1^ *Re-analysis of published data presented by M.Boelaert at the TDR/WHO expert meeting: Visceral Leishmaniasis (VL) Transmission Dynamics and Continued Sources of Infection during the Post-VL Elimination Phase (Maintenance Phase) in the Indian Sub-continent, Kathmandu, Nepal, from 30 March to 1 April 2016*.

**Table K: Data presented at the TDR-WHO expert meeting on cost-effective VL case detection and vector control for the support of the VL elimination initiative in the Indian sub-continent, Freiburg, Germany (23 – 25 November 2015)**

**
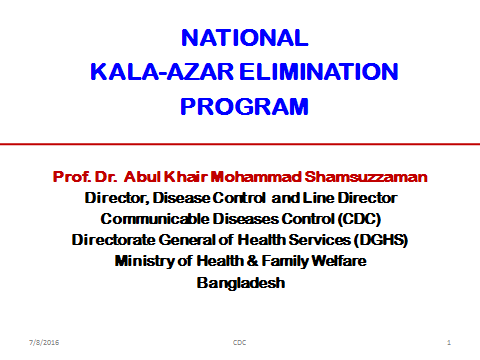

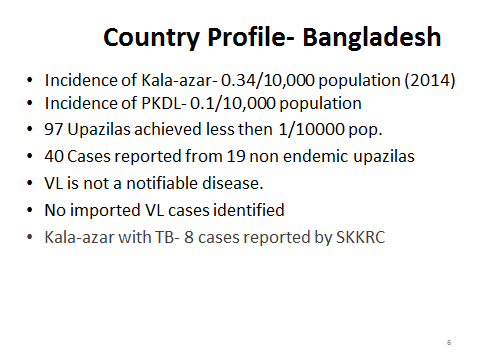
**

**Table L: Modelling transmission of Leishmania donovani infection in the Indian subcontinent** (Parameters estimated by the model are shaded grey)

|  | **Mubayi (2010)**  [[54](#_ENREF_54)] | **Stauch (2011)**  [[55](#_ENREF_55)] | **Stauch (2012)**  [[56](#_ENREF_56)] | **Stauch (2014)**  [[57](#_ENREF_57)] | **Le Rutte (2016)**  [[58](#_ENREF_58)] | **Medley (2015)**  [[59](#_ENREF_59)] | **Chapman (2015)**  [[60](#_ENREF_60)] |
| --- | --- | --- | --- | --- | --- | --- | --- |
| **Objectives** | - Quantify levels of under-reporting | - Effects of treatment and SF control on VL elimination | - Explain why antimonial treatment failure rates increased in Bihar, India | - Effects of SF control on elimination | - Potential of ALI and PKDL as reservoirs of infection | - Effects of early diagnosis on VL elimination | - Estimate durations of different disease stages and ALI to VL progression rate |
| **Model structure** | - Four transmission stages (susceptible, latent, infected, recovered);  - Three transmission stages in SF (susceptible, latent, infected); | - Five transmission stages (susceptible, early and late asymptomatic, early and late recovered);  - Four clinical stages (untreated symptomatic, 1^st^ line treated, 2^nd^ line treated, PKDL);  - Three transmission stages in SF (susceptible, latent, infected); | Same as Stauch 2011;  - additional set of parameters for infection with resistant parasites; | Same as Stauch 2011 | Similar to Stauch 2011  - Model 1: early and late ALI, VL and PKDL are infective;  - Model 2: reactivation of infection after initial infection;  - Model 3: only VL and PKDL are infective; | - Four health seeking stages (fever non-health seeking, fever health seeking, VL non-health seeking, VL health seeking);  - Three host transmission stages (susceptible, latent infected, dormant) | - Multi-state Markov model with 5 different states (susceptible, asymptomatic, symptomatic VL, recovered and dead) |
| **Main differences between models** | - Separate pathway for treatment in private and public facilities;  - Recovered stage does not lead back to susceptible stage; | - Allows ALI (but not untreated VL) to putatively recover and develop PKDL; | Same as Stauch 2011;  - Model extended by stages representing infections with parasites resistant to antimonial treatment; | Same as Stauch 2011;  - Model parameters used to calculate thresholds for R_e_; | Same as Stauch 2011;  - Age-specific mortality rate and age-dependent exposure to SF biting;  - Assumptions vary about reservoirs of infection;  - Allows untreated VL (but not ALI) to putatively recover and develop PKDL; | - Health seeking behaviour added to transmission model;  - Does not include explicit SF population;  - Does not include a separate PKDL transmission component; | - Probabilistic model with movement between disease states described by transition intensities  - SF population not modelled  - PKDL not included |
| **Data used to fit the model** | District level VL surveillance records | KALANET trial [[33](#_ENREF_33), [61](#_ENREF_61)] | Twelve clinical studies [[62](#_ENREF_62)] | Same as Stauch 2011 | Same as Stauch 2011 | India, Nepal [[63](#_ENREF_63)]; Bangladesh (unpublished data) | Data from Mymensingh district, Bangladesh [1] |
| **Assumptions (SF)** | - homogenous mixing;  - SF population size constant;  - SF density (no. of SF/human) assumed constant; | | | | - Same as for other models;  - no excess mortality among infected SF; | - Same as for other models (implicit) | -- |
| **Assumptions (host)** | - homogenous mixing within but not between districts; | - Susceptible human population size constant  - infectiveness of host increases monotonically from time of infection to late asymptomatic infection  - susceptible gets infected at a rate that depends on SF biting rate, no. of SF per human, and infectiveness of SF;  - VL deaths are negligible | | | | Same as Stauch 2011  - Transmission parameters same across Bangladesh, India and Nepal;  - Health seeking behaviour varies between countries | - Transition rates between disease states constant in time and space, but vary with age, sex and bed net use |
| **SF parameters** | | | | | | | |
| Life span | 14dy | 14dy | 14dy | 14dy | 14dy | -- | -- |
| Latent period | 5dy | 5dy | 5dy | 7.8dy | 5dy | -- | -- |
| Feeding interval | -- | 4dy | 4dy | 5.6dy | 4dy | -- | -- |
| Prevalence of infected SF |  | 0.5% | 0.5% | 0.5% | 0.927% (India); 0.5% (Nepal) |  |  |
| No. of SF / human |  | 5.27 | 5.27 | 7.34 | 0.075 – 0.269 (India);  0.038 – 0.172 (Nepal); |  |  |
| Infectiveness of SF |  | 100% | 100% | 100% | 100% | -- | -- |
| **Host parameters** | | | | | | | |
| Life expectancy | 60y | 40y | 40y | 40y |  | 50y | -- |
| Birth rate |  |  |  |  | 21/1000 pop |  | -- |
| Death rate |  |  |  |  | Age specific |  | Age- and disease-state-specific |
| Excess MR in untreated VL |  | 0.2/mo | 0.2/mo | 0.2/mo | 0.25/mo (fitting); 0.2/mo (predicting); |  |  |
| **Duration of transmission stages** | | | | | | | |
| Susceptible |  | 150dy | 150dy | 150dy |  |  |  |
| Latent | 4mo |  |  |  |  | 80dy |  |
| Fever onset to seeking care |  |  |  |  |  | country specific parameter value generated; |  |
| Fever onset to VL |  |  |  |  |  | 33 – 55dy |  |
| VL symptoms to seeking care |  |  |  |  |  | 5dy |  |
| VL treatment to dormant |  |  |  |  |  | Immediate |  |
| Early ALI |  | 60dy | 60dy | 60dy | 382 – 387dy |  | 147dy |
| Late ALI |  | 12dy | 12dy | 12dy | 133 – 138dy |  |  |
| Infective | 3.8mo |  |  |  |  |  |  |
| Untreated VL |  |  |  |  | 30dy (fitting);  45dy (predicting); |  | 140dy |
| 1^st^ treatment |  |  |  |  | 30dy (fitting);  2.5dy (predicting); |  |  |
| 2^nd^ treatment |  |  |  |  | 30dy (fitting);  10dy (predicting); |  |  |
| Early recovered |  | 74dy | 74dy | 74dy | 482 - 490dy |  | ~3y |
| Late recovered |  | 307dy | 307dy | 315dy | 2 or 5y (pre-set) |  |  |
| Time to PKDL |  | 21mo | 21mo | 21mo | 5 or 15y (pre-set) |  |  |
| Treatment (private) | 1.33 times that in public sector |  |  |  |  |  |  |
| Treatment (public) | 23dy |  |  |  |  |  |  |
| **Prevalence of transmission stages** | | | | | | | |
| Susceptible |  | 26% | 26% | 26% |  |  | 53-55% |
| Early ALI |  | 10% | 10% | 10% |  |  | 7-10% |
| Late ALI |  | 2% | 2% | 2% |  |  |  |
| VL |  | 0.015% | 0.015% | 0.015% |  |  | 0-1% |
| PKDL |  | 0.005% | 0.005% | 0.005% |  |  | -- |
| Early recovered |  | 12% | 12% | 12% |  |  | 38% |
| Late recovered |  | 50% | 50% | 50% |  |  |  |
| **Proportion transiting to different transmission stages** | | | | | | | |
| Prop ALI recovered |  | 99.77% | 99.77% | 99.77% |  |  |  |
| Prop ALI developing VL |  | 0.33% | 0.33% | 0.28% | 2.8 – 3.9% | 4% | 14.7% |
| Prop ALI developing PKDL |  | 0.01% | 0.01% | 0.01% |  |  |  |
| Prop VL spontaneously recovered |  |  |  |  | 0.03% |  |  |
| Prop VL developing PKDL |  |  |  |  | 5% (pre-set) |  |  |
| Prop seeking care before VL symptoms |  |  |  |  |  | 76 – 87% (Bihar);  14 – 49% (Nepal);  88 – 93% (Bangladesh); |  |
| Treatment failure rate |  |  | 5 – 100% (varied) |  | 5% |  | 2.7% |
| Basic reproduction number (R_0_) | 1.3 – 2.1; | 3.94 |  | 4.71 |  | Varied: 0.5 – 5 |  |
| **Infectiveness of different transmission stages** | | | | | | | |
| Infectiveness of early ALI |  | Half of infectiveness of late ALI | Half of late ALI infectiveness | Half of late ALI infectiveness | Half of late ALI infectiveness | 0.1% of clinical VL or varied |  |
| Infectiveness of late ALI |  | 2.5% | 2.5% | 2.5% | 2.88 – 3.32% | 10% of clinical VL or varied |  |
| Infectiveness of VL |  | 100% | 100% | 100% | 100% | Not modelled explicitly |  |
| Infectiveness of VL on treatment |  |  |  |  | 50% | 0% |  |
| Infectiveness of PKDL |  | 100% | 100% | 100% | 50% (model 1,2);  2.32 – 2.72 (model 3); | Very low |  |
| **Scenarios tested** | Not applicable | - Varying treatment duration of VL and PKDL;  - Early or late VL case detection;  - Varying treatment fatality;  - Varying treatment failure;  - Varying relapse rate; | - Varying pathogenicity of resistant parasite;  - Varying virulence of resistant parasite;  - Varying transmissibility of ALI or VL;  - Varying transmissibility of SF; | Reduction of SF density:  - IRS (by killing SF);  - LLIN (by killing SF);  - EVM (by reducing breeding capacity); | - Optimal IRS (SF density reduced by 63%);  - Sub-optimal IRS (SF density reduced by 31.5%);  IRS in three VL endemic settings; | - Reduced delay in diagnosis;  - Diagnosed and treated early during stage of non-specific febrile symptoms; | Not applicable |
| **Main conclusion** | - Under-reporting most sensitive to changes in VL incidence; | - Vector based interventions reduced the prevalence (and incidence) of asymptomatic and symptomatic VL  – Interventions should be long enough to detect and effectively treat PKDL; | Increased transmissibility of resistant parasite (and not antimonial resistance alone) most likely reason to explain high treatment failure rate seen between 1980 – 1997 in Bihar; | - SF control (not early detection and treatment of VL patients) can eliminate VL  - SF control combined with active case detection and treatment to prevent resurgence; | - VL elimination depends on assumptions about the main reservoir of infection (asymptomatic, reactivation in recovered, or PKDL); | - Reduced delay in diagnosis affects short term reduction in VL cases, but unlikely to eliminate VL in long term;  - Resurgence if delays in diagnosis returns; | - ALI may contribute significantly to transmission even if much less infectious than symptomatic VL cases due to their long infection period and much larger numbers  - Further studies required to measure asymptomatic infectivity; |
| **Limitations of model** | - Spatial clustering not considered  - Lifelong immunity assumed for treated VL;  - Latent stage equivalent to incubation period and assumed to be non-infective period; | - Spatial clustering not considered  - Does not allow untreated VL to putatively recover to and proceed to develop PKDL;  - Loss of LST positivity (immunity) has to be assumed together with a prevalence of 50% LST+ pop. as host population turnover is not sufficient to yield prevalence of 26% susceptible  - Does not consider the potential of domestic animals as alternative reservoirs by reducing the SF biting rate on human; | Same as Stauch 2011; | Same as Stauch 2011;  - Assumes that the effect of LLIN on R_e_ is only thru reduction in SF density by killing SF – the LLIN effect on Re (reducing transmission) by reducing contact (biting) rate not considered; | Same as Stauch 2011;  - Model solution possible only when duration of late recovered stage was less than 7y – however this parameter pre-set to 2 or 5y was reasonable; | - Spatial clustering not considered  - Does not consider infectiveness potential of asymptomatic and PKDL;  Dormant stage infectiousness is an average of potentially highly infectious PKDL and non-infectious treated VL;  - Does not consider SF dynamics in transmission; | - Spatial clustering not considered  - Infection rate constant in time and independent of numbers of asymptomatic and symptomatic VL cases;  - Role of SFs in transmission dynamics ignored;  - Imperfect sensitivity and specificity of diagnostic tests not accounted for; |

Note: SF (sandfly); VL (visceral leishmaniasis); ALI (asymptomatic leishmania infection); PKDL (post kala-azar dermal leishmaniasis); R_e_ (effective reproduction number); MR (mortality rate); R_0_ (basic reproduction number); IRS (indoor residual spraying); LLIN (long lasting insecticide nets); EVM (environmental management for vector control);

**References**

1. Bern, C., et al., *The epidemiology of visceral leishmaniasis and asymptomatic leishmanial infection in a highly endemic Bangladeshi village.* Am J Trop Med Hyg, 2007. **76**(5): p. 909-14.

2. Bimal, S., et al., *Usefulness of the direct agglutination test in the early detection of subclinical Leishmania donovani infection: a community-based study.* Ann Trop Med Parasitol, 2005. **99**(8): p. 743-9.

3. Burza, S., et al., *Five-year field results and long-term effectiveness of 20 mg/kg liposomal amphotericin B (Ambisome) for visceral leishmaniasis in Bihar, India.* PLoS Negl Trop Dis, 2014. **8**(1): p. e2603.

4. Burza, S., et al., *Risk factors for visceral leishmaniasis relapse in immunocompetent patients following treatment with 20 mg/kg liposomal amphotericin B (Ambisome) in Bihar, India.* PLoS Negl Trop Dis, 2014. **8**(1): p. e2536.

5. Burza, S., et al., *Visceral leishmaniasis and HIV co-infection in Bihar, India: long-term effectiveness and treatment outcomes with liposomal amphotericin B (AmBisome).* PLoS Negl Trop Dis, 2014. **8**(8): p. e3053.

6. Burza, S., et al., *Post Kala-Azar dermal leishmaniasis following treatment with 20 mg/kg liposomal amphotericin B (Ambisome) for primary visceral leishmaniasis in Bihar, India.* PLoS Negl Trop Dis, 2014. **8**(1): p. e2611.

7. Gidwani, K., et al., *Longitudinal seroepidemiologic study of visceral leishmaniasis in hyperendemic regions of Bihar, India.* Am J Trop Med Hyg, 2009. **80**(3): p. 345-6.

8. Gidwani, K., et al., *Persistence of Leishmania donovani antibodies in past visceral leishmaniasis cases in India.* Clin Vaccine Immunol, 2011. **18**(2): p. 346-8.

9. Hasker, E., et al., *Latent infection with Leishmania donovani in highly endemic villages in Bihar, India.* PLoS Negl Trop Dis, 2013. **7**(2): p. e2053.

10. Singh, S.P., et al., *The epidemiology of Leishmania donovani infection in high transmission foci in India.* Trop Med Int Health, 2010. **15 Suppl 2**: p. 12-20.

11. Sudarshan, M., et al., *Quantitative PCR in epidemiology for early detection of visceral leishmaniasis cases in India.* PLoS Negl Trop Dis, 2014. **8**(12): p. e3366.

12. Sudarshan, M. and S. Sundar, *Parasite load estimation by qPCR differentiates between asymptomatic and symptomatic infection in Indian visceral leishmaniasis.* Diagn Microbiol Infect Dis, 2014. **80**(1): p. 40-2.

13. Huda, M.M., et al., *Low prevalence of Leishmania donovani infection among the blood donors in kala-azar endemic areas of Bangladesh.* BMC Infect Dis, 2013. **13**: p. 62.

14. Islam, M.Z., et al., *ELISA with recombinant rKRP42 antigen using urine samples: a tool for predicting clinical visceral leishmaniasis cases and its outbreak.* Am J Trop Med Hyg, 2012. **87**(4): p. 658-62.

15. Kumar, R., et al., *Enzyme-linked immunosorbent assay for recombinant K39 antigen in diagnosis and prognosis of Indian visceral leishmaniasis.* Clin Diagn Lab Immunol, 2001. **8**(6): p. 1220-4.

16. Maurya, R., et al., *Evaluation of PCR for diagnosis of Indian kala-azar and assessment of cure.* J Clin Microbiol, 2005. **43**(7): p. 3038-41.

17. Ostyn, B., et al., *Failure of miltefosine treatment for visceral leishmaniasis in children and men in South-East Asia.* PLoS One, 2014. **9**(6): p. e100220.

18. Rijal, S., et al., *Increasing failure of miltefosine in the treatment of Kala-azar in Nepal and the potential role of parasite drug resistance, reinfection, or noncompliance.* Clin Infect Dis, 2013. **56**(11): p. 1530-8.

19. Singh, S., V. Kumari, and N. Singh, *Predicting kala-azar disease manifestations in asymptomatic patients with latent Leishmania donovani infection by detection of antibody against recombinant K39 antigen.* Clin Diagn Lab Immunol, 2002. **9**(3): p. 568-72.

20. Sinha, P.K., et al., *Effectiveness and safety of liposomal amphotericin B for visceral leishmaniasis under routine program conditions in Bihar, India.* Am J Trop Med Hyg, 2010. **83**(2): p. 357-64.

21. Sinha, P.K., et al., *Liposomal amphotericin B for visceral leishmaniasis in human immunodeficiency virus-coinfected patients: 2-year treatment outcomes in Bihar, India.* Clin Infect Dis, 2011. **53**(7): p. e91-8.

22. Burza, S., et al., *HIV and visceral leishmaniasis coinfection in Bihar, India: an underrecognized and underdiagnosed threat against elimination.* Clin Infect Dis, 2014. **59**(4): p. 552-5.

23. Topno, R.K., et al., *Asymptomatic infection with visceral leishmaniasis in a disease-endemic area in bihar, India.* Am J Trop Med Hyg, 2010. **83**(3): p. 502-6.

24. Uranw, S., et al., *Post-kala-azar dermal leishmaniasis in Nepal: a retrospective cohort study (2000-2010).* PLoS Negl Trop Dis, 2011. **5**(12): p. e1433.

25. Vallur, A.C., et al., *Biomarkers for intracellular pathogens: establishing tools as vaccine and therapeutic endpoints for visceral leishmaniasis.* Clin Microbiol Infect, 2014. **20**(6): p. O374-83.

26. Ansari, N.A., P. Sharma, and P. Salotra, *Circulating nitric oxide and C-reactive protein levels in Indian kala azar patients: correlation with clinical outcome.* Clin Immunol, 2007. **122**(3): p. 343-8.

27. Ostyn, B., et al., *Transmission of Leishmania donovani in the Hills of Eastern Nepal, an Outbreak Investigation in Okhaldhunga and Bhojpur Districts.* PLoS Negl Trop Dis, 2015. **9**(8): p. e0003966.

28. Bhattarai, N.R., et al., *PCR and direct agglutination as Leishmania infection markers among healthy Nepalese subjects living in areas endemic for Kala-Azar.* Trop Med Int Health, 2009. **14**(4): p. 404-11.

29. Bhattarai, N.R., et al., *Domestic animals and epidemiology of visceral leishmaniasis, Nepal.* Emerg Infect Dis, 2010. **16**(2): p. 231-7.

30. Hasker, E., et al., *Strong association between serological status and probability of progression to clinical visceral leishmaniasis in prospective cohort studies in India and Nepal.* PLoS Negl Trop Dis, 2014. **8**(1): p. e2657.

31. Khanal, B., et al., *Serological markers for leishmania donovani infection in Nepal: Agreement between direct agglutination test and rK39 ELISA.* Trop Med Int Health, 2010. **15**(11): p. 1390-4.

32. Ostyn, B., et al., *Incidence of symptomatic and asymptomatic Leishmania donovani infections in high-endemic foci in India and Nepal: a prospective study.* PLoS Negl Trop Dis, 2011. **5**(10): p. e1284.

33. Picado, A., et al., *Effect of village-wide use of long-lasting insecticidal nets on visceral Leishmaniasis vectors in India and Nepal: a cluster randomized trial.* PLoS Negl Trop Dis, 2010. **4**(1): p. e587.

34. Picado, A., et al., *Risk factors for visceral leishmaniasis and asymptomatic Leishmania donovani infection in India and Nepal.* PLoS One, 2014. **9**(1): p. e87641.

35. Rijal, S., et al., *Epidemiology of Leishmania donovani infection in high-transmission foci in Nepal.* Trop Med Int Health, 2010. **15 Suppl 2**: p. 21-8.

36. Singh, R.P., et al., *Post-kala-azar dermal leishmaniasis (PKDL) in visceral leishmaniasis-endemic communities in Bihar, India.* Trop Med Int Health, 2012.

37. Srivastava, P., et al., *Molecular and serological markers of Leishmania donovani infection in healthy individuals from endemic areas of Bihar, India.* Trop Med Int Health, 2013. **18**(5): p. 548-54.

38. Joshi, A.B., et al., *Chemical and environmental vector control as a contribution to the elimination of visceral leishmaniasis on the Indian subcontinent: cluster randomized controlled trials in Bangladesh, India and Nepal.* BMC Med, 2009. **7**: p. 54.

39. Singh, U.K., et al., *Miltefosine in children with visceral leishmaniasis: a prospective, multicentric, cross-sectional study.* Indian J Pediatr, 2006. **73**(12): p. 1077-80.

40. Bern, C., et al., *Loss of leishmanin skin test antigen sensitivity and potency in a longitudinal study of visceral leishmaniasis in Bangladesh.* Am J Trop Med Hyg, 2006. **75**(4): p. 744-8.

41. Das, V.N., et al., *Clinical epidemiologic profile of a cohort of post-kala-azar dermal leishmaniasis patients in Bihar, India.* Am J Trop Med Hyg, 2012. **86**(6): p. 959-61.

42. Garg, V.K., et al., *Post-kala-azar dermal leishmaniasis in Nepal.* Int J Dermatol, 2001. **40**(3): p. 179-84.

43. Gurubacharya, R.L., et al., *Prevalence of visceral leishmania & HIV co-infection in Nepal.* Indian J Med Res, 2006. **123**(3): p. 473-5.

44. Koirala, S., et al., *Epidemiological study of kala-azar by direct agglutination test in two rural communities of eastern Nepal.* Trop Med Int Health, 2004. **9**(4): p. 533-7.

45. Kumar, R., et al., *Sero-epidemiological study of kala-azar in a village of Varanasi district, India.* Trop Med Int Health, 2006. **11**(1): p. 41-8.

46. Mondal, D., et al., *Enhanced case detection and improved diagnosis of PKDL in a Kala-azar-endemic area of Bangladesh.* PLoS Negl Trop Dis, 2010. **4**(10).

47. Nandy, A., A.B. Neogy, and A.B. Chowdhury, *Leishmanin test survey in an endemic village of Indian kala-azar near Calcutta.* Ann Trop Med Parasitol, 1987. **81**(6): p. 693-9.

48. Rahman, K.M., et al., *Increasing incidence of post-kala-azar dermal leishmaniasis in a population-based study in Bangladesh.* Clin Infect Dis, 2010. **50**(1): p. 73-6.

49. Rai, R.N., et al., *Clinico-epidemiological profiles of post-kala-azar dermal leishmaniasis in Varanasi.* J Commun Dis, 1989. **21**(3): p. 214-7.

50. Saha, S., et al., *Visceral leishmaniasis is preventable in a highly endemic village in West Bengal, India.* Trans R Soc Trop Med Hyg, 2009. **103**(7): p. 737-42.

51. Saran, R., A.K. Gupta, and M.C. Sharma, *Evidence of Leishmania donovani infection in household members residing with visceral leishmaniasis patients.* J Commun Dis, 1992. **24**(4): p. 242-4.

52. Schenkel, K., et al., *Visceral leishmaniasis in southeastern Nepal: a cross-sectional survey on Leishmania donovani infection and its risk factors.* Trop Med Int Health, 2006. **11**(12): p. 1792-9.

53. Singh, N., et al., *Animal reservoirs of visceral leishmaniasis in India.* J Parasitol, 2013. **99**(1): p. 64-7.

54. Mubayi, A., et al., *Transmission dynamics and underreporting of Kala-azar in the Indian state of Bihar.* J Theor Biol, 2010. **262**(1): p. 177-85.

55. Stauch, A., et al., *Visceral leishmaniasis in the Indian subcontinent: modelling epidemiology and control.* PLoS Negl Trop Dis, 2011. **5**(11): p. e1405.

56. Stauch, A., et al., *Treatment of visceral leishmaniasis: model-based analyses on the spread of antimony-resistant L. donovani in Bihar, India.* PLoS Negl Trop Dis, 2012. **6**(12): p. e1973.

57. Stauch, A., et al., *Model-based investigations of different vector-related intervention strategies to eliminate visceral leishmaniasis on the Indian subcontinent.* PLoS Negl Trop Dis, 2014. **8**(4): p. e2810.

58. Le Rutte, E.A., et al., *Feasibility of eliminating visceral leishmaniasis from the Indian subcontinent: explorations with a set of deterministic age-structured transmission models.* Parasit Vectors, 2016. **9**(1): p. 24.

59. Medley, G.F., et al., *Health-seeking behaviour, diagnostics and transmission dynamics in the control of visceral leishmaniasis in the Indian subcontinent.* Nature, 2015. **528**(7580): p. S102-8.

60. Chapman, L.A., et al., *Quantification of the natural history of visceral leishmaniasis and consequences for control.* Parasit Vectors, 2015. **8**: p. 521.

61. Picado, A., et al., *Longlasting insecticidal nets for prevention of Leishmania donovani infection in India and Nepal: paired cluster randomised trial.* BMJ, 2010. **341**: p. c6760.

62. Olliaro, P.L., et al., *Treatment options for visceral leishmaniasis: a systematic review of clinical studies done in India, 1980-2004.* Lancet Infect Dis, 2005. **5**(12): p. 763-74.

63. Boettcher, J.P., et al., *Visceral leishmaniasis diagnosis and reporting delays as an obstacle to timely response actions in Nepal and India.* BMC Infect Dis, 2015. **15**: p. 43.
